# Nanoscale Degradation Study of a Commercial Pt–Co/C Fuel-Cell Electrocatalyst at Elevated Temperature Utilizing Identical-Location Scanning Transmission Electron Microscopy

**DOI:** 10.1021/acs.jpcc.5c03548

**Published:** 2025-08-19

**Authors:** Ante Matošin, Lazar Bijelić, Ana Rebeka Kamšek, Goran Dražić, Matija Gatalo, Marjan Bele, Nejc Hodnik

**Affiliations:** † National Institute of Chemistry, Hajdrihova 19, 1001 Ljubljana, Slovenia; ‡ Faculty of Chemistry and Chemical Technology, Večna pot 113, 1000 Ljubljana, Slovenia; § University of Nova Gorica, Vipavska 13, 5000 Nova Gorica, Slovenia; ∥ ReCatalyst, Hajdrihova 19, 1001 Ljubljana, Slovenia; ⊥ Institute of Metals and Technology, Lepi pot 11, 1000 Ljubljana, Slovenia

## Abstract

Understanding the degradation mechanisms of Pt-based alloy electrocatalysts under realistic operating conditions, such as elevated temperature, is essential for improving the durability of proton exchange membrane fuel cells (PEMFCs). This study investigates the degradation behavior of a commercial PEMFC Pt–Co/C electrocatalyst on the individual nanoparticle scale, employing identical location scanning transmission electron microscopy (IL-STEM), in combination with electrochemical methods. The catalyst was subjected to the modified US Department of Energy protocol at an elevated temperature (fast potential cycling between 0.6 and 0.95 V_RHE_ with a 3 s hold at each potential limit for 10,000 cycles in 0.1 M HClO_4_ at 60 °C) in order to partially simulate real-world operating conditions. To evaluate the specific role of temperature in the degradation process, additional experiments were carried out at room temperature. The primary aim was to elucidate temperature-dependent nanostructural changes and correlate them with electrochemical characterization. Results reveal distinct alterations in Pt–Co alloy nanoparticles’ morphology, such as necking and increased circularity. These are driven by surface energy minimization via coalescence, dissolution, and redeposition mechanisms. By correlating nanoscale observations with changes in the intrinsic electrochemical properties, our study provides crucial insights into the degradation pathways at elevated temperatures, informing the design of more durable catalyst formulations for future fuel cell devices.

## Graphical abstract

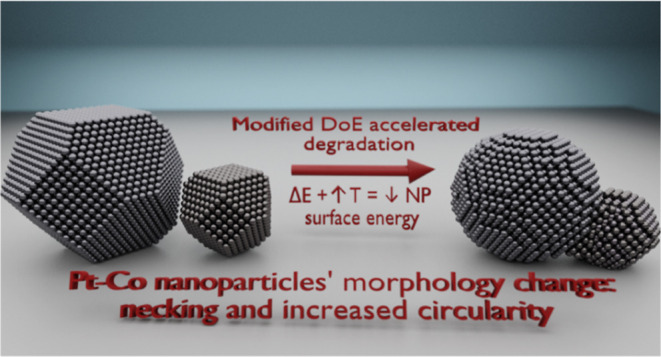

## 1 Introduction

Large-scale commercial viability of proton exchange membrane fuel cell (PEMFC) technology as an eco-friendly alternative for internal combustion engines relies on several critical factors. One of them is the cost of production, which heavily depends on the quantity of Pt used in the PEMFC catalyst, especially for the inhibited cathodic reaction (oxygen reduction).^1^ Another is the long-term stability and retention of performance in operational conditions. There are also numerous other conditions that have to be met, including policies, technologies, and infrastructure necessary for affordable hydrogen production and storage,^2,3^ however, the focus of this paper is specifically on the electrocatalyst activity and stability.

There have been numerous studies concerned with increasing the utilization of Pt and even using nonprecious metal group alternatives.^4,5^ However, due to its intrinsic properties, the electrocatalysts currently used at production scale for automotive PEMFCs are still based on Pt.^6,7^ The first step toward higher utilization was to disperse Pt nanoparticles on a conductive and stable support with a high surface area, mostly using various carbon black materials (e.g., Ketjen Black, Vulcan).^8^ The next promising approach has been to alloy Pt with a less noble metal (M).^9^ This is most commonly done with 3d transition metals, primarily Co, Cu, Fe, or Ni.^10,11^ In addition to the dilution of Pt atoms, these nanoalloys exhibit better activity compared to pure Pt. The state-of-the-art explanation for this higher activity is given through interrelated structural and/or electronic effects, namely strain, ligand, and ensemble effects.^12–17^ The influence of these effects is evident in the change of the binding strength, that is, the adsorption and desorption of reactants, reaction intermediates, products, and spectator species, which in turn governs the kinetics of the oxygen reduction reaction (ORR).^18^

However, with the increased activity of the Pt alloy electrocatalysts comes lower intrinsic stability. This is to be expected, as the less noble 3d transition metals are inherently unstable and prone to dissolution at the operational conditions of the PEMFC.^19,20^ Through dissolution, newly introduced transition metal ions decrease the performance by blocking the Pt surface,^21^ reducing proton transport by depositing in the membrane^22^ and catalyzing reactions that produce detrimental species for the fuel cell, e.g., the Fenton reaction.^23^ Although Pt is significantly more stable than 3d transition metals, it also dissolves due to low pH and oxidizing voltage characteristic of PEMFCs. Pt dissolution is mostly present through the transient dissolution mechanism induced by the formation and reduction of Pt oxide,^24,25^ which in turn leads to other degradation processes, such as Ostwald ripening^26^ and formation of Pt bands in the membrane.^27,28^ The critical role of temperature on Pt and M dissolution, but also on the redeposition of Pt, has been studied by Cherevko and D̵ukić et al.^29,30^ By utilizing an electrochemical flow cell linked to an inductively coupled plasma mass spectrometer (EFC-ICP–MS) methodology, the potential and time-resolved dissolution signals of Pt and less noble metal M measured at different temperatures pointed to an increase in Pt/M dissolution with the temperature increase. Moreover, since much higher dissolution of M (which follows Pt dissolution) was observed compared to Pt with the increasing temperature, it is assumed that Pt redeposition also increases with temperature, contributing to the masking of the Pt dissolution signal. Still, it is not clear how Pt and less noble metal dissolution behave at the nanoscale level upon degradation that includes elevated temperatures.

Aside from Pt and M dissolution, the catalyst also degrades through the corrosion of its carbon support. This is largely observed through electrochemical oxidation to CO_2_, while the presence of Pt and elevated temperatures (≈80 °C) increase the rate of the reaction.^31–33^ Carbon corrosion is especially pronounced during the start-up/shutdown conditions when the fuel cell can reach potentials as high as 1.6 V_NHE_.^34^ Similar to the dissolution of Pt and the alloying metal, carbon corrosion can also lead to secondary degradation mechanisms, such as agglomeration of previously separated neighboring Pt-alloy nanoparticles.^35^

Fundamental understanding of these degradation mechanisms is crucial for improving the stability of Pt-alloy electrocatalysts. One of the biggest challenges of studying and developing these materials is to find a compromise between gathering relevant information about the intrinsic properties, using the least amount of material, and the simplest methods within an acceptable time frame. In order to precisely simulate operating conditions and study long-term degradation behavior of the electrocatalyst, accelerated degradation tests (ADTs) are performed in membrane electrode assemblies (MEAs). They commonly follow the United States Department of Energy (DoE) degradation protocol, which consists of 30,000 trapezoidal wave cycles in a voltage range between 0.6 and 0.95 V, with a 3 s hold at potential limits, at 80 °C, 150 kPa absolute pressure and relative humidity of 100%.^36^ This protocol aims to simulate operational conditions spanning a 5000 h lifetime.^37^ There are, however, disadvantages to using this method. It is very energy and time-consuming, and it can be challenging to separate the influence of the numerous parameters and components within the system, complicating the interpretation of the data. To tackle these problems, several methods using aqueous half-cells for ADTs have been developed, often based on the existing rotating disk electrode setups.^30,38,39^

Aside from determining electrochemical properties and measuring their decrease, aqueous half-cell methods can be modified to be used in combination with identical location scanning transmission electron microscopy (IL-STEM) to observe structural changes in the electrocatalyst nanoparticles.^40,41^ The application of IL-STEM for the investigation of fuel cell catalyst degradation has been pioneered by the Mayrhofer group,^42^ and has been since utilized by many others. Among these, Prof. Strasser and his group are notable for using this methodology in their research. Regarding ORR, their group has reported a number of studies exploring the impact of electrocatalyst morphology and composition on activity. By implementing IL-STEM, they demonstrated Ni dissolution^43^ and shape degradation of doped octahedral PtNi/C particles after an ADT, explained by detailed microstructural studies of the atomic rearrangement processes on the surfaces of the samples.^44^ Nanoscale structural properties, such as composition, morphology, exposed facets, defects and strain, govern the electrocatalytic activity of Pt-based nanoalloy materials.^45–47^ Therefore, to improve long-term stability, understanding of the local changes in structural properties induced by operational conditions is vital.

It is important to note that, although the methods based on aqueous half-cells result in more straightforward and easily interpretable characterization, the acquired data do not fully replicate the conditions encountered in real PEMFC operation. That is to say, the electrocatalyst’s intrinsic properties and degradation processes observed in simplified aqueous electrolyte setups do not directly translate to an MEA stack. These methods represent a trade-off between time-and-material-consuming MEA stability tests and less close-to-real but faster RDE stability tests. This, however, does not mean that characterizations utilizing such methods are irrelevant. It simply means that these methods and results should primarily serve as an initial screening tool to identify promising electrocatalyst candidates, which can later be tested more precisely in closer-to-real conditions. Moreover, by thorough comparison of results gained from aqueous half-cells and MEAs, it is possible to correlate certain properties and processes through the optimization of parameters in the former in order to more closely describe the behavior of the catalyst in the latter.^48^ In this manner, Takahashi and Kocha described the influence of different electrolytes on the determination of activity and stability of a Pt/C sample, showing that perchloric acid has the lowest anion adsorption on Pt and simulates catalytic activity closest in magnitude to values obtained in MEAs using Nafion membrane.^49^ Moreover, in regards to Pt dissolution, Gilbert et al. used data on the particle size distribution after degradation in both aqueous half-cell and MEA and concluded that the setup with a flow-through electrolyte at room temperature can be used to mimic MEA degradation.^50^ When it comes to IL-STEM, particularly, Dr. David Cullen and his group have tackled the recreation of fuel cell catalyst degradation in aqueous environments, showing how these setups can be tailored to better match the degree and type of degradation observed in fuel cell experiments. Specifically, a study done by Yu et al. demonstrated that changing the potential window and increasing the concentration of Pt ions in the solution helps simulate fuel cell degradation mechanisms in aqueous half-cells more precisely.^41^

Within this study, IL-STEM images of a commercial Pt–Co/C electrocatalyst were taken before and after the ADT, employing an in-house designed high temperature disk electrode (HT-DE) setup to perform a modified DoE degradation protocol. This was done to partially simulate PEMFC operational conditions and analyze the degradation behavior of the sample in a reliable and timely manner. Combining the IL-STEM and HT-DE methodologies, we were able to observe nanoscale changes in the electrocatalyst, including a variety of degradation phenomena resulting from the electrochemical protocol and elevated temperature. Additionally, electrochemical characterization was performed on electrocatalyst thin films using the same HT-DE setup to perform the degradation protocol, and a standard rotating disk electrode setup to determine the intrinsic properties before and after the ADTs. Degradation protocols for both IL-STEM and electrochemical characterization were done at 60 °C and room temperature (RT) to observe the influence of increased temperature on nanostructural changes and losses in intrinsic properties.

## 2 Experimental Section

Degradation behavior of a commercial dealloyed Pt–Co/C electrocatalyst (Umicore Elyst Pt50 0690) was studied using the following methods.

### 2.1 Accelerated Degradation Tests via High Temperature Disk Electrode (HT-DE) and Thin-Film Rotating Disk Electrode (TF-RDE) Methodology

#### 2.1.1 Setup

ADTs on electrocatalyst thin films were performed in a setup previously used by our group.^30,38,51^ It is composed of a double-wall glass cell, with hot distilled water (≈60 °C) circulating from the thermostat (MGW Lauda) through the outer wall, heating the 0.1 M HClO_4_ electrolyte (Carl Roth, Rotipuran Supra) inside the cell.

A conventional three-electrode system was utilized, consisting of a reversible hydrogen electrode (HydroFlex) as the reference, a graphite rod counter electrode and a glassy carbon disk (Pine Instruments) with a geometric surface area of 0.196 cm^2^ as the working electrode. The electrodes were connected to a SP-200 Potentiostat (Biologic). To keep the electrolyte level in the cell constant, in addition to the utilization of a Liebig condenser, open parts of the HT-DE cell were covered by in-house designed polyether ether ketone (PEEK) caps.

Electrochemical characterization, that is, oxygen reduction reaction (ORR) polarization curves and CO electrooxidation cyclic voltammograms, were measured in a thin-film rotating disk electrode (TF RDE) setup before and after ADTs (Figure 1). Measurements were conducted with an SP-200 Potentiostat (Biologic) in a two-compartment electrochemical cell with an identical three-electrode system.

**Figure 1:**
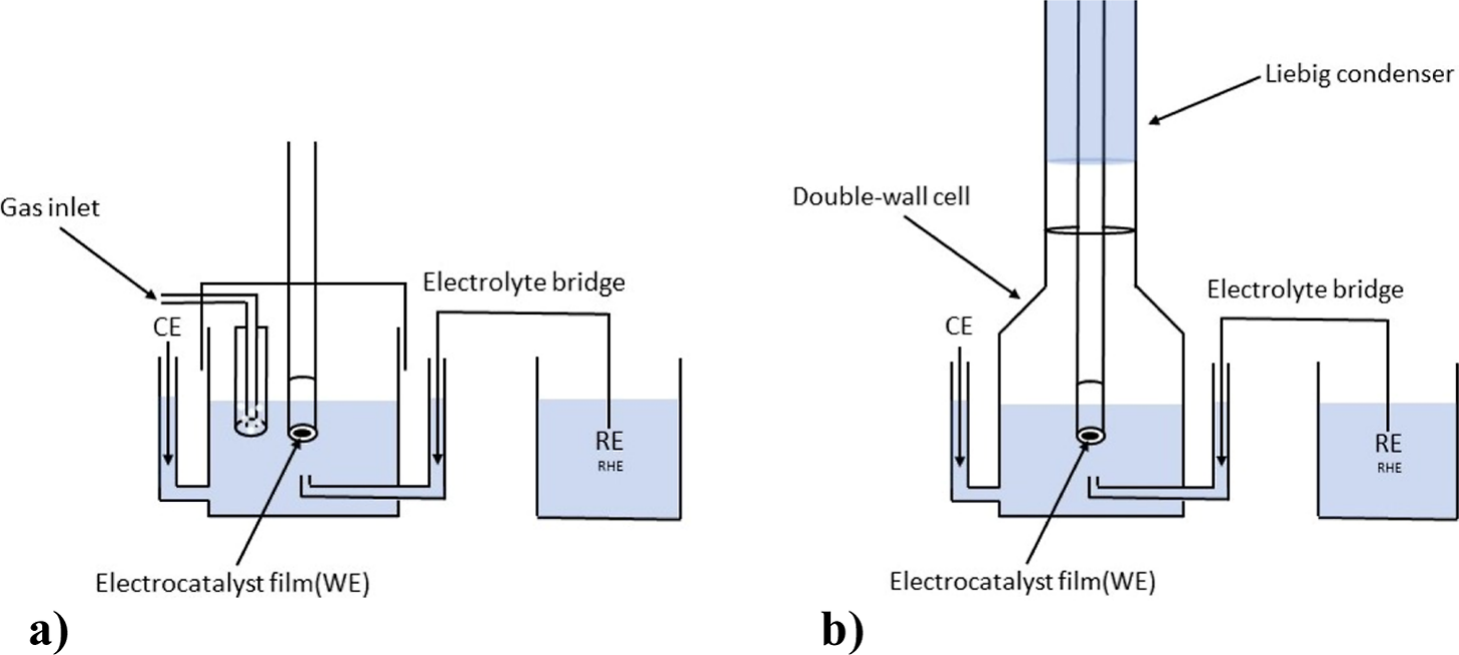
Schematic depiction of (a) RDE and (b) HT-DE setups.

#### 2.1.2 Procedure

To prepare the thin films, 20 μL of 1 mg mL^–1^ water-based ultrasonicated (ASonic) electrocatalyst ink was pipetted onto the glassy carbon electrode (Pine Instruments) and dried under ambient conditions. Such preparation resulted in an electrocatalyst loading of 20 μg, or rather 0.102 mg_catalyst_ cm^–2^. After the film had dried, 5 μL of Nafion solution (ElectroChem) diluted in isopropanol (1:50) was added. The electrode was then mounted on a rotator (Pine Instruments) and submerged in 0.1 M HClO_4_ saturated with N_2_. The electrocatalyst first underwent cyclic voltammetry spanning 50 cycles from 0.05 to 1.2 V_RHE_, with a scan rate of 300 mV s^–1^ and under a 600 rpm rotation rate. This was done to remove potential traces of Co remaining on the Pt-rich surface of the core–shell nanoparticle after chemical activation. After exchanging the electrolyte, ORR polarization curves were measured under oxygen saturation with 1600 rpm in the potential window 0.05–1.0 V_RHE_ with a 20 mV s^–1^ scan rate. At the end of the ORR measurement, the electrolyte was saturated with CO during a potential hold at 0.05 V_RHE_ to ensure successful CO adsorption. Afterward, the remaining CO in the electrolyte was purged with N_2_. CO electrooxidation was performed using the same potential window and scan rate as in ORR, but without rotation.

Next, the working electrode was carefully transferred to the HT-DE setup and the ADT was performed using the same SP-200 Potentiostat (Biologic). The protocol was composed of 10,000 trapezoidal wave cycles from 0.6 to 0.95 V_RHE_ at 60 °C in 0.1 M HClO_4_. After the last cycle, the working electrode was carefully transferred back to the TF-RDE setup, and the ORR polarization curve, as well as CO electrooxidation, were measured once again. This was followed by another 50 cycles of cyclic voltammetry, and another round of ORR and CO electrooxidation measurements (same parameters as previously described). This was done to make sure that there were no contaminants on the thin film during the final measurements.

The electrochemically active surface area (ECSA) was determined by integrating the charge from CO electrooxidation. For the ORR, specific activity (SA) and mass activity (MA) were calculated at 0.95 V_RHE_, with 85% compensated ohmic resistance.^52,53^

Detailed reasoning behind the protocol modifications can be found in the work done by D̵ukić et al.^25^ Briefly, the reason behind reducing the temperature from 80 to 60 °C has been to minimize the evaporation of the 0.1 M HClO_4_ electrolyte during such long-lasting experiments. As part of our group’s prior research,^30,54^ it was shown that the effect of temperature already has a significant impact at around 50 °C; thus, a balance between minimizing evaporation, as well as having a large enough impact on the durability, has been chosen as the most sensible approach.

Electrochemical characterization was done only before and after each ADT experiment (ex-situ), as opposed to periodic measurements during the protocol, since past research has revealed that frequent periodic measurements during the ADTs result in additional degradation of the Pt-alloy cathodes, thus influencing the overall results.^55^ Furthermore, seeing as the DoE proposed ADT protocol is relatively slow in terms of throughput, even when using our modified high-temperature liquid half-cell setup, the number of cycles was reduced from 30,000 to 10,000 cycles. In the previously mentioned work done by D̵ukić et al.,^25^ a comparison between ADTs consisting of different numbers of cycles was given, and it was concluded that sufficient data on the degradation can be gained even after 10,000 cycles.

### 2.2 Identical Location Scanning Transmission Electron Microscopy (IL-STEM)

To track the morphological changes occurring at the same catalyst location, as-purchased Pt–Co/C nanoparticles were first deposited on a gold TEM grid (Agar Scientific, Holey Carbon Films on 300 mesh Gold). To do this, 50 μL of 1 mg mL^–1^ suspension was diluted in 0.5 mL of Milli-Q water and 0.45 mL of isopropanol. After brief ultrasonication of the diluted suspension in the ultrasound bath (ASonic), 5 μL of the suspension was pipetted on a gold TEM grid and dried under ambient conditions. Afterward, IL-STEM images of the electrocatalyst sample before the ADT were taken on a Cs probe-corrected scanning transmission electron microscope (JEOL ARM 200 CF) at 80 kV. Several spots were identified on the TEM grid and imaged at different magnifications. The regions of interest (ROI) selected for the identical location imaging were kept under a reduced electron dose in order to avoid/minimize any induced modification with the beam. Severe electron exposure, such as beam alignment and elemental mapping with energy dispersive X-ray spectroscopy (EDX), was not performed on the selected IL-STEM spots. After the STEM imaging, a modified glassy carbon disk electrode (Figure 2b) was employed to hold the gold TEM grid containing the electrocatalyst sample during the ADT. A droplet of 0.1 M HClO_4_ was first pipetted onto the glassy carbon, which was then followed by careful placing of the TEM grid onto the droplet. Threads were machined at the tip of the electrode so that a hollow PEEK cap could be screwed on. By doing so, the TEM grid comes into direct contact with the glassy carbon disk electrode. Furthermore, the cap was made with an opening at the top to facilitate electrolyte contact with the electrocatalyst sample on the TEM grid. Once the modified glassy carbon electrode holding the gold TEM grid was assembled, it was transferred to the same HT-DE setup used for thin film stability analysis, and the same electrochemical protocol was applied. When the protocol was finished, the modified glassy carbon electrode was disassembled, and the TEM grid was removed and dried at ambient temperature. IL-STEM images of the electrocatalyst sample after the ADT were then taken in the previously described manner.

**Figure 2:**
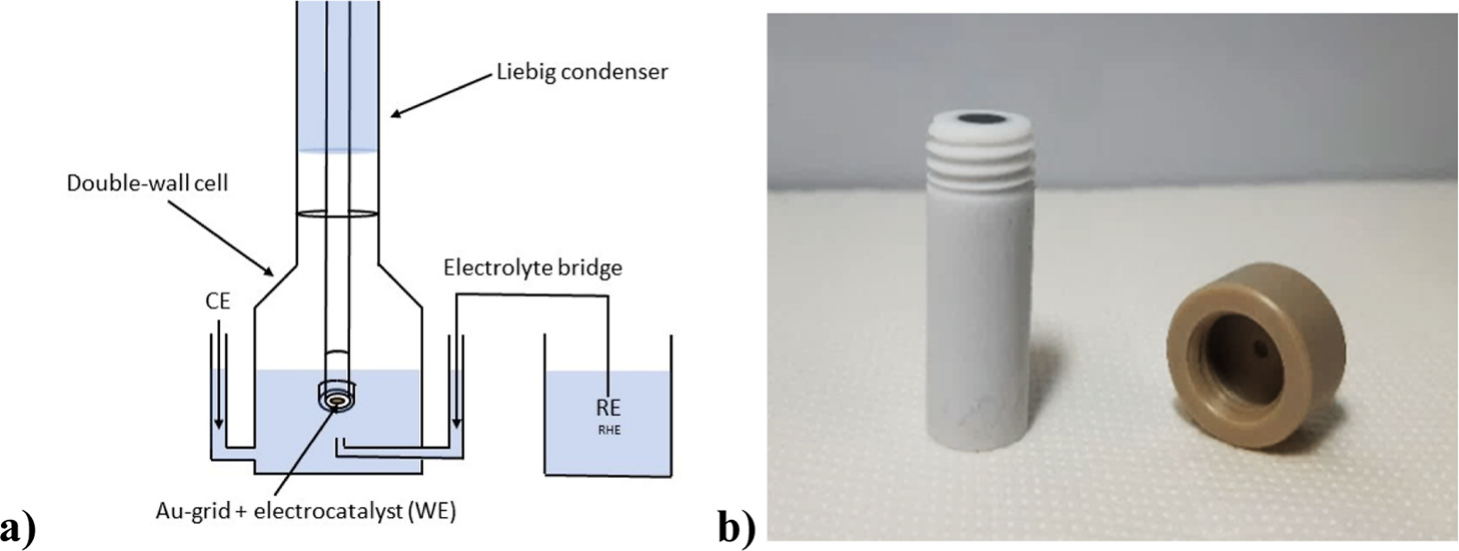
(a) HT-DE setup with the gold STEM grid; (b) threaded glassy carbon disk electrode with the hollow PEEK cap.

### 2.3 STEM Image Analysis

Nanoparticles in IL-STEM images were analyzed to provide insights into size and shape changes. Only nanoparticles that were in focus and did not touch the edges of the images were considered. They were annotated manually to produce particle masks using the Supervisely platform.^56^ Each mask was defined by its equivalent sphere diameter and circularity, calculated from the measured particle areas and perimeters as

$$diameter=\frac{perimeter}{\pi }$$

$$circularity=4\pi \left(\frac{area}{{perimeter}^{2}}\right)$$

Particle masks from images taken at identical locations were connected to form events, which allowed us to track each annotated nanoparticle. Only events consisting of a single mask per image were considered (i.e., no detachments, attachments, or agglomerations). A total of 80 such events were captured after the ADT performed at 60 °C, while 58 events were captured at RT. For all particles, we determined and visualized changes in size and shape as histograms to analyze the overall variations in size and circularity distributions. The particle analysis code was developed using the Python programming language.

### 2.4 Scanning Electron Microscopy Energy-Dispersive X-ray (SEM–EDX) Analysis

SEM–EDX analysis was performed using a detector (SDD Ultim max 100, Oxford, UK) at 20 kV. The sample was prepared using the following procedure: a small amount of powder electrocatalyst (1–3 mg) was put on a 13 mm polished metal disk and covered with a metal disk of the same size. The sample was pelleted with a manual press until a pellet with a thickness of about 50 μm was obtained. Standard SEM pin mounts (Agar Scientific) covered with conductive carbon tape (Agar Scientific) were used to hold the pellet.

### 2.5 XRD Analysis

The powder X-ray diffraction (XRD) measurements of the commercial Pt–Co/C and Pt/C electrocatalyst samples were carried out on a PANalytical X’Pert PRO diffractometer with Cu Kα radiation (λ = 1.541874 Å) in the 2θ range from 10 to 60° with a 0.039° step per 300 s using a fully opened Pixcel detector. The sample was prepared on zero-background Si holders.

### 2.6 XRF Analysis

For precise determination and consistency of electrocatalyst Pt loading for electrochemical characterization, X-ray fluorescence measurements (XRF), using the Thermo Fisher Scientific ARL QUANT’X EDXRF with SDD500 silicon drift detector, were performed on thin films deposited on glassy carbon electrodes. For this purpose, special holders for the electrodes were designed and 3D printed (Bambu Lab X1-Carbon) using PLA filaments. Electrocatalyst thin film Pt loadings were measured in an air atmosphere with a Pd medium filter at 20 kV and under rotation before each electrochemical measurement.

## 3 Results and Discussion

In this paper, a commercial Pt–Co/C electrocatalyst from Umicore (Elyst Pt50 0690), with a 41.8 wt % Pt and 5.5 wt % Co loading (determined using the SEM–EDX and XRF) was investigated. The focus of the study was on the nanoscale degradation mechanisms of the Pt–Co/C and the influence of temperature on those mechanisms. Therefore, IL-STEM images of the Pt–Co/C sample were taken before and after the ADTs performed at 60 °C and RT. What we first noticed by comparing low-magnification imaging (Supporting Information Figures S1 and S2) before and after degradation at 60 °C is that the sample appeared relatively stable, as very small changes could be seen. The carbon support outline remained mostly unchanged, suggesting only minor carbon corrosion during the ADT. This is not surprising due to the low upper potential limit used in our ADT. At higher magnifications, however, we observed more substantial alterations in the structure of the electrocatalyst nanoalloy particles. In Figure 3a,b, identical location images depict two examples of Pt–Co nanoparticles partially dissolving, but also becoming connected with a narrow bridge, a mechanism sometimes referred to as “necking”. In this case, necking occurred at the Pt {111} facet (Supporting Information Figure S5). A potential explanation for this mechanism is that it represents an intermediate stage during the coalescence of two particles, which includes oriented attachment.^57^ In order for the mechanism to occur, particles need to be sufficiently close. Matching of the exposed facets and the orientation of the nanoparticle are additional parameters that could affect the rate of coalescence.

**Figure 3:**
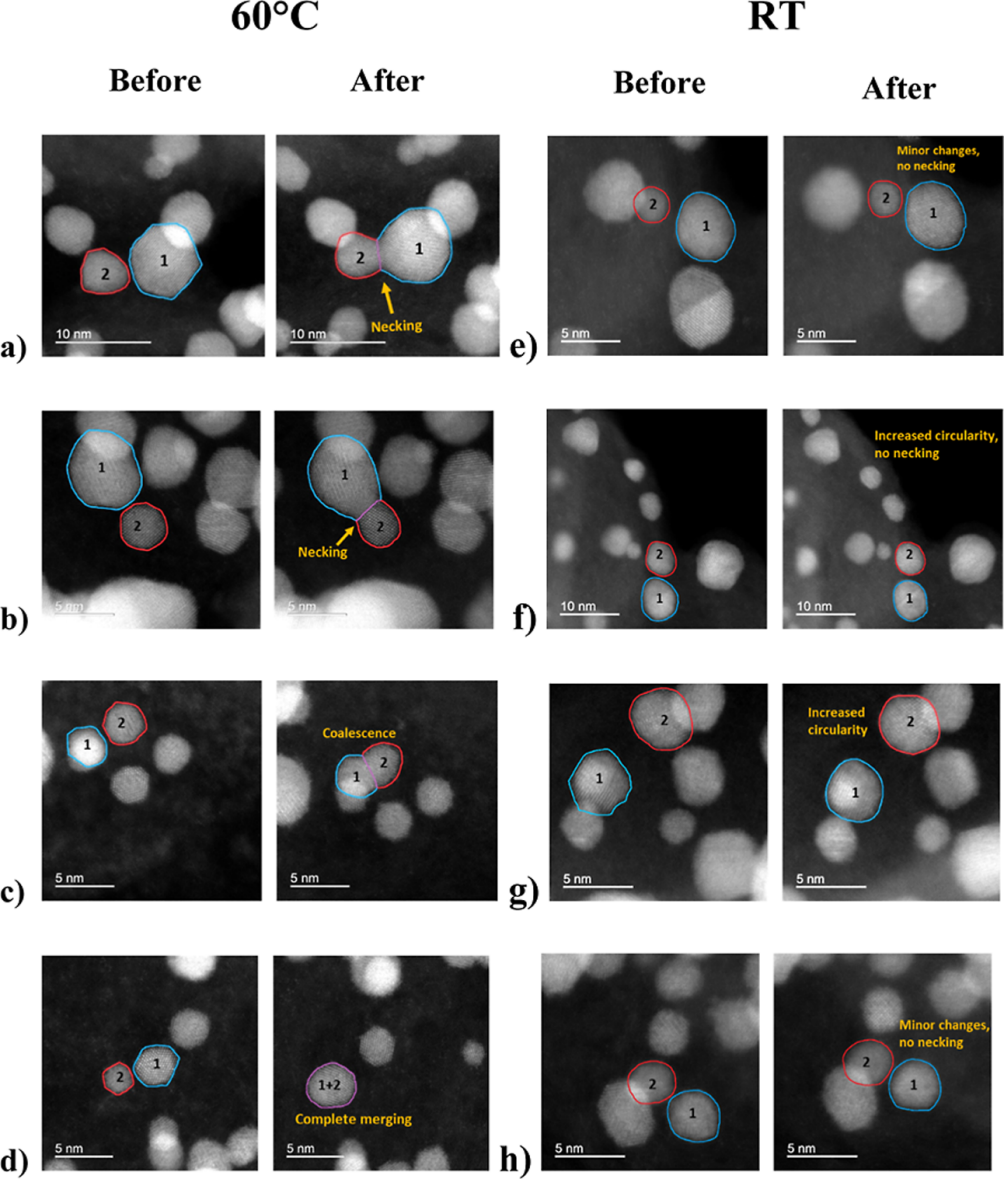
High-angle annular dark field (HAADF) IL-STEM images of the Umicore Pt–Co/C sample (Elyst Pt50 0690), after the ADT performed at 60 °C, depicting “necking” (a,b) and later stages of coalescence (c,d). Additional images of the electrocatalyst (e–h) were taken after the degradation protocol performed at RT. The protocols were performed in 0.1 M HClO_4_ by potential cycling between 0.6 and 0.95 V_RHE_ for 10,000 cycles.

In this particular case, it is still unclear if it is induced by dissolution and redeposition of Pt from the connecting or neighboring particles, atomic diffusion influenced by temperature/electrochemical protocol, or a combination of both. From the IL-STEM images taken after the ADT performed at 60 °C, it can be seen that the merging of the nanoparticles is accompanied by distinct morphological changes. As described by Ouyang et al., the attachment of two nanoparticles results in a highly negative surface curvature around the contact point, which makes the structure energetically unstable.^58^ The high surface energy drives the tendency for the minimization of the net surface area, inducing mass transport to fill the region around the contact point and culminating in the formation of a “neck”.^59^ This mechanism is described extensively in papers concerning the synthesis of metal nanoparticles, e.g. the synthesis of gold nanoparticles utilizing ultrasonic spray pyrolysis described by Shariq et al.^60^ Although in this particular paper, the morphology changes after synthesis were induced by the electron beam overexposure on gold particles, parallels can still be drawn with the changes induced by the ADT on Pt–Co nanoalloy. Regarding Pt-based electrocatalysts specifically, Gatalo et al. reported coalescence of Pt–Cu_3_ nanoparticles during thermal annealing, with evidence of the necking mechanism observed via IL-STEM.^61^ Similarly, Chattot et al. observed necking in a 40 wt % Pt/C electrocatalyst integrated into an MEA during ADTs, which became more pronounced following additional cyclic voltammetry.^62^ In their paper, they describe an increase in coherent domain size observed after cycling to low electrode potentials, suggesting a rapid coalescence of aggregated nanoparticles, triggered by the reduction of Pt oxides.

According to previous research, coalescence can be divided into four stages: (1) movement and rotation of the metal NPs; (2) necking mechanism; (3) oriented attachment; (4) reshaping of the metal NPs.^60^ During coalescence, the metal NPs wiggle and/or reorient to align with similar facets from other nearby NPs.^63^ At the same time, “necking” takes place, which facilitates the coalescence of the metal NPs. Apart from the interparticle distance, facets and orientation, an additional parameter that could play a role is the nanoparticle size, or more precisely, size distribution. Previous studies describe the process occurring primarily between similarly sized nanoparticles, with a diameter of approximately 4 nm or less.^64^ In the case of the Pt–Co/C images in Figure 3a,b, necking occurred between one bigger (*d*_1a_ = 7.65 nm; *d*_1b_ = 7.14 nm) and one smaller (*d*_2a_ = 4.97 nm, *d*_2b_ = 3.80 nm) particle.

In Figure 3c,d, additional stages of coalescence can be seen. The third image pair (Figure 3c) depicts the oriented attachment process slightly before the total merging and final reshaping. In this case, all of the particles involved are of similar size, or rather, they have a similar diameter (*d* ≈ 3.5 nm). The fourth pair of images (Figure 3d) shows two separated nanoparticles becoming completely merged into one larger, more spherical particle after the ADT. In this case, a slightly bigger nanoparticle shifts toward the smaller, and likely vice versa, until they merge.

In the case of images taken after the ADT at RT (Figure 3e–h), no coalescence was observed, but instances of restructuring toward higher sphericity were still present, although less pronounced than at 60 °C. A closer inspection of the images presented in Figure 4a–d reveals that the sharp edges and corners of the nanoparticles, highlighted in blue, gradually disappear, resulting in a more rounded morphology after degradations at both temperatures, but seemingly more pronounced at 60 °C. This transformation is likely driven by the system’s tendency to minimize surface energy. As a fundamental property of metallic nanoparticles, surface energy plays a crucial role in both crystal synthesis and stability. During synthesis, surface energy and consequently growth rates are modified by the utilization of capping agents to achieve a variety of polyhedral nanoparticle shapes (cubic, octahedral, cuboctahedral, truncated octahedra, etc.).^65^ In the case of Pt, which has a face-centered cubic (fcc) crystal structure, these polyhedral shapes are often enclosed by a mix of {111}, {110} and {100} facets. For an fcc single crystal, the surface energies, γ, associated with the low-index crystallographic planes are in the order of γ_(111)_ < γ_(100)_ < γ_(110)_.^66^

**Figure 4:**
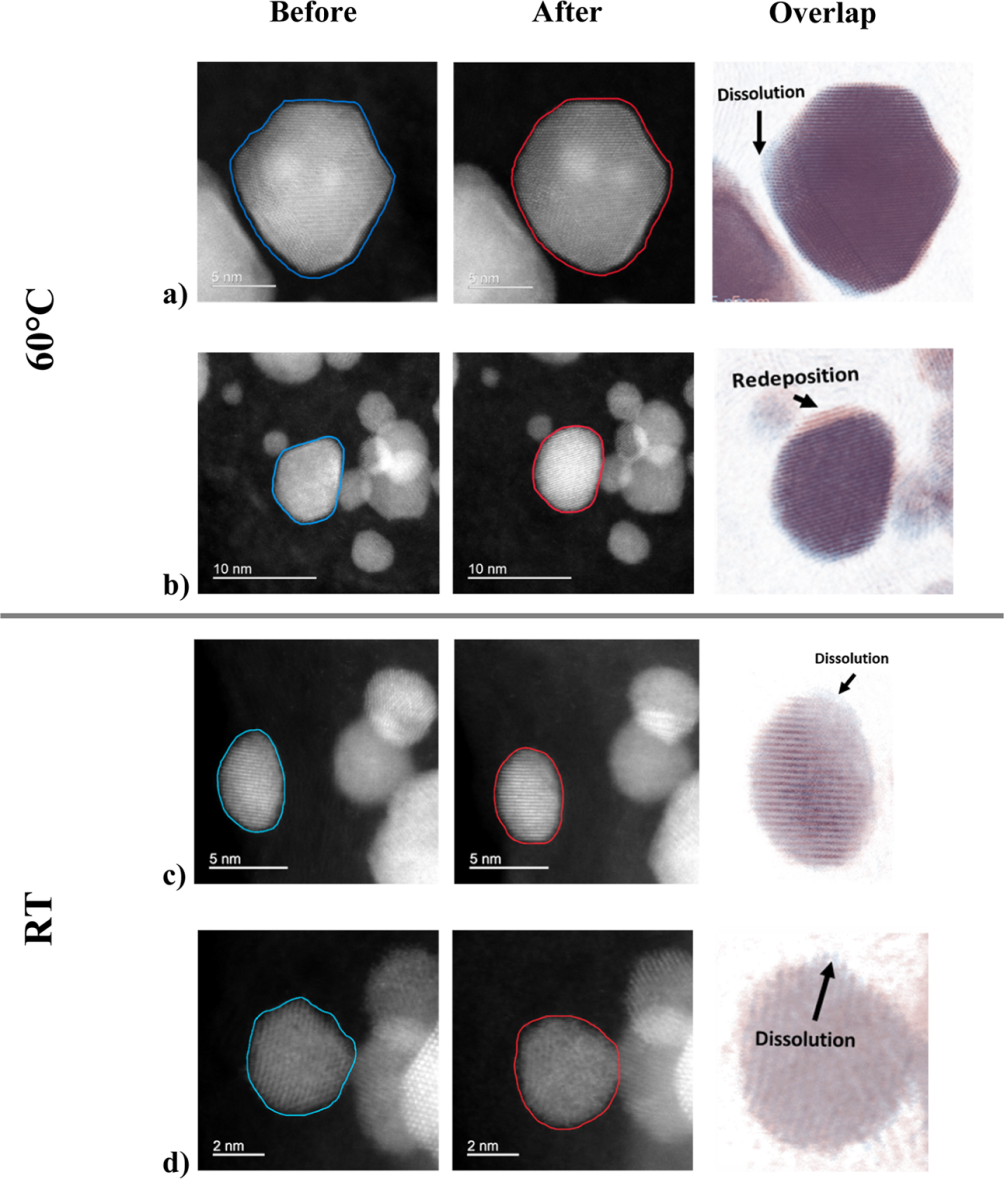
High-angle annular dark field (HAADF) IL-STEM images of the Umicore Pt–Co/C sample (Elyst Pt50 0690) depicting dissolution and redeposition influenced by surface energy after the ADT at 60 °C (a,b) and RT (c,d). The third image in each row represents the overlap of the two bright field images of the same nanoparticle before (blue) and after (red) the ADT.

From the XRD diffraction patterns shown in Figure 5, according to the presence of five obvious peak maxima, we conclude the presence of phases *P*4/*mmm* ^67^ and *Fm* 3̅ *m*.^68^ Furthermore, based on the ratios of the peak intensities, we estimate that approximately 80% of the Pt–Co volume is *Fm* 3̅ *m*, while 20% is *P*4/*mmm*. We cannot rule out the presence of a small quantity of the *Pm*3*m* phase, but we also cannot reliably confirm its presence.

**Figure 5:**
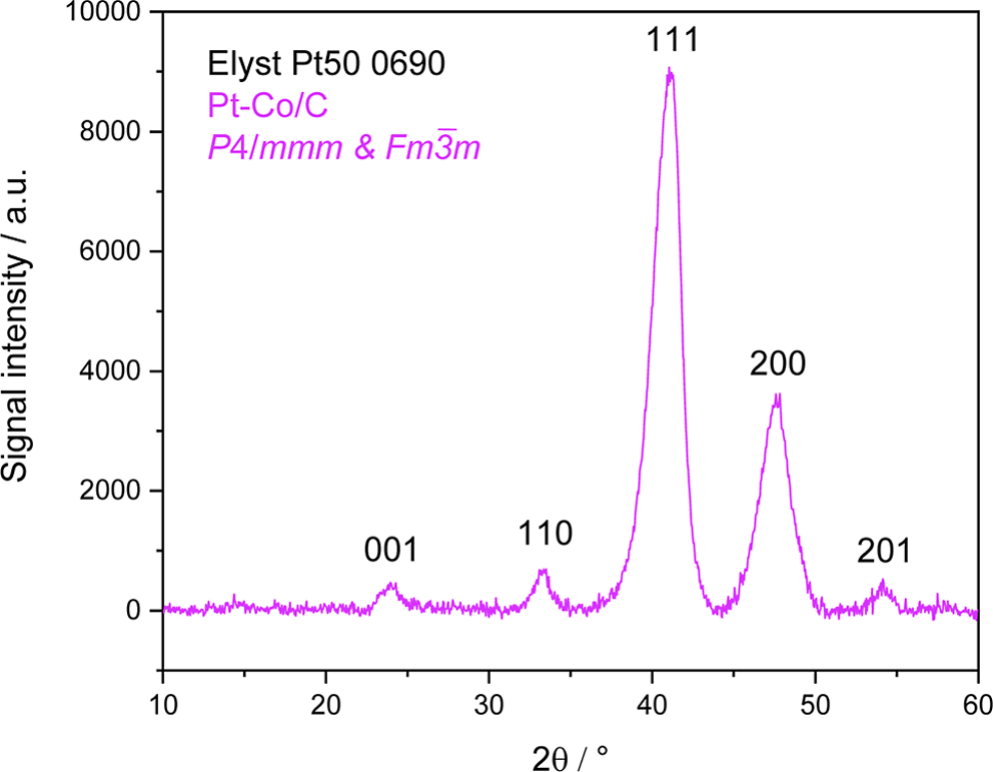
XRD diffraction patterns measured from the Umicore Pt–Co/C sample (Elyst Pt50 0690).

The theory for determining the equilibrium shape of a crystal based on the principle of minimizing surface free energy was first introduced by Wulff^69^ and subsequently refined in later works by Dinghas and Herring.^70,71^ From the viewpoint of Wulff construction and taking into consideration surface/volume ratio, one of the energetically most favored well-defined crystal shapes for nanometric size Pt particles would be a truncated octahedron, whose surface has eight close-packed {111} and six square {100} facets.^72^ However, the preparation of catalysts with dispersed metal particles of well-defined and uniform shapes is not possible on a commercial scale, which is why different polyhedra and geometrically irregular shapes can be seen in practice. Although electrocatalyst nanoparticles with well-defined morphology exhibit a greatly enhanced activity, it is not long-lived, as it is very difficult to preserve these particle shapes under operational conditions.^73^ As shown in Figure 4a–d, particles transform to more thermodynamically stable, round-like shapes under oxide formation/reduction cycling.

This was confirmed through the size and circularity analysis of the nanoparticles in IL-STEM images after ADTs performed at 60 °C and RT. During the analysis, only the nanoparticles that were in focus and did not touch the edges of the images were considered. As can be seen from the visual circularity/diameter mapping and histograms in Figure 6a, there is a clear trend of increasing circularity after the performed ADT at 60 °C, with the average diameter of analyzed nanoparticles changing by up to 0.5 nm, and with the majority of particles becoming smaller. Based on the IL-STEM images, both dissolution and redeposition^30^ seem to be contributing toward this increase in circularity. The influence of dissolution toward increased circularity can be seen in Figure 4a–d in the overlap of nanoparticle images before (blue) and after (red) the ADT. Pt atoms from the sharp and protruding edges dissolve, and the particle becomes more rounded following the ADT. On the other hand, during the cathodic cycling, Pt redeposits on more concave surfaces in order to achieve a lower surface energy. This is probably most evident while observing the nanoparticle in Figure 4b. Considering the flat top segment of the particle outline before the ADT, it would seem that the most energetically favorable process to reach increased circularity, and consequently lower surface energy, was the redeposition of Pt atoms, which are colored red in the overlap image. The size and circularity analysis following the ADT at RT (Figure 6b) also indicates a trend toward increased circularity, albeit to a significantly lesser extent than that observed at 60 °C. These subtle structural changes, along with the absence of coalescence, are evident in Figure 3e–h and 4c,d, and are further supported by electrochemical measurements.

**Figure 6:**
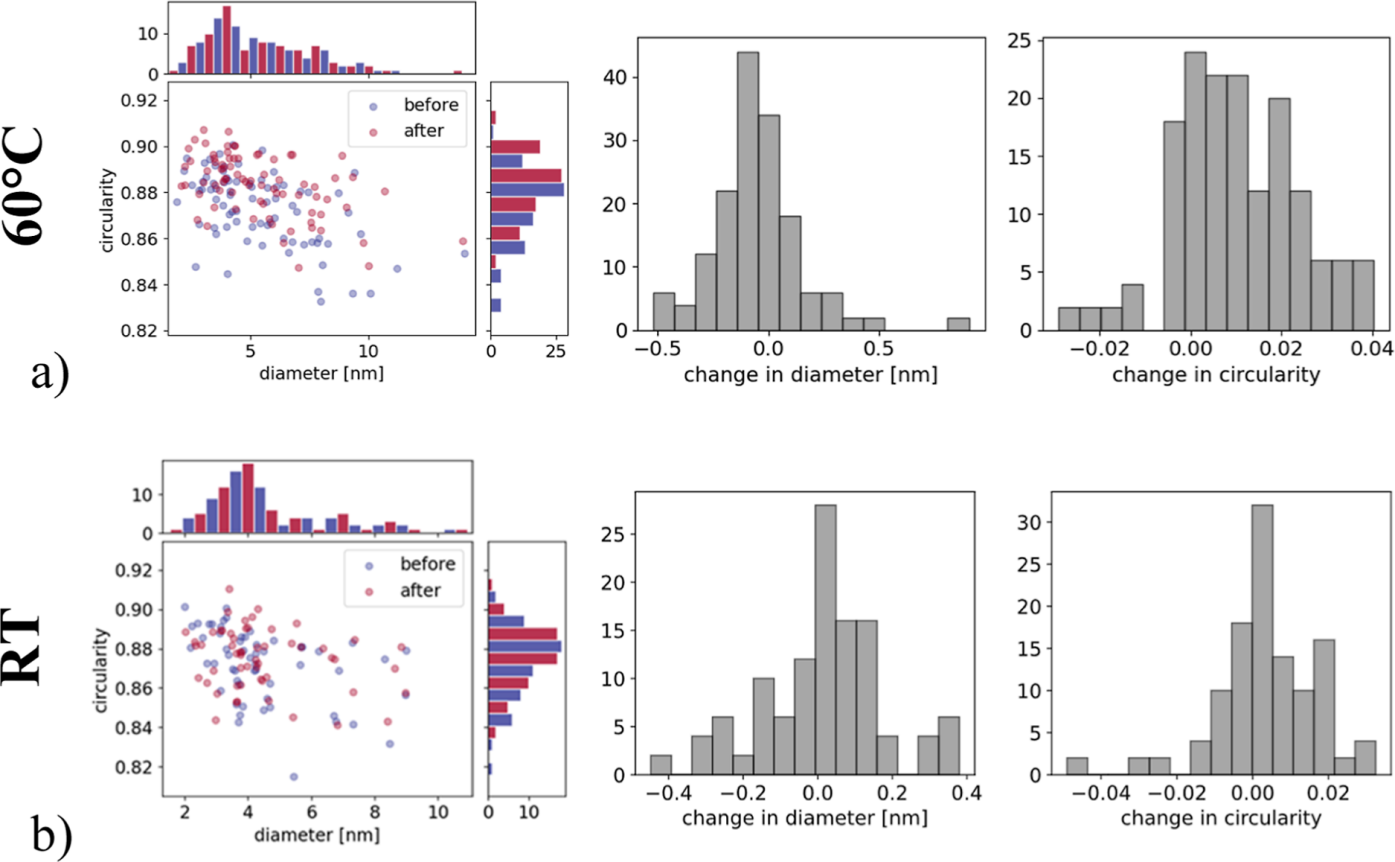
Size and circularity analysis before and after the ADTs at 60 °C and RT. Changes in size and shape were determined and visualized through histograms to analyze the overall variations in size and circularity distributions.

Electrochemical characterization of the commercial Pt–Co/C sample consisted of first doing cyclic voltammetry for 50 cycles (Supporting Information Figure S12a), followed by measuring ORR and CO electrooxidation in a TF-RDE setup. Next, an ADT was performed for 10,000 cycles, at 60 °C or RT, lasting approximately 19.5 h. Then, two more sets of ORR and CO electrooxidation measurements were performed, with 50 cycles of CV in between (Supporting Information Figure S12b). The additional cycling was performed to double-check the presence of potential contaminants on the thin film surface following the ADT. Evidence of Pt–Co/C degradation at 60 °C can be seen from the ORR curves (Figure 7a) in the curve shift toward more negative potentials. Degradation is also seen in the Tafel plot (Supporting Information Figure S12e) through the decrease in kinetic current density. However, the slope of the Tafel curve has remained the same (≈60 mV dec^–1^), meaning that the rate-determining reaction step was unchanged.

**Figure 7:**
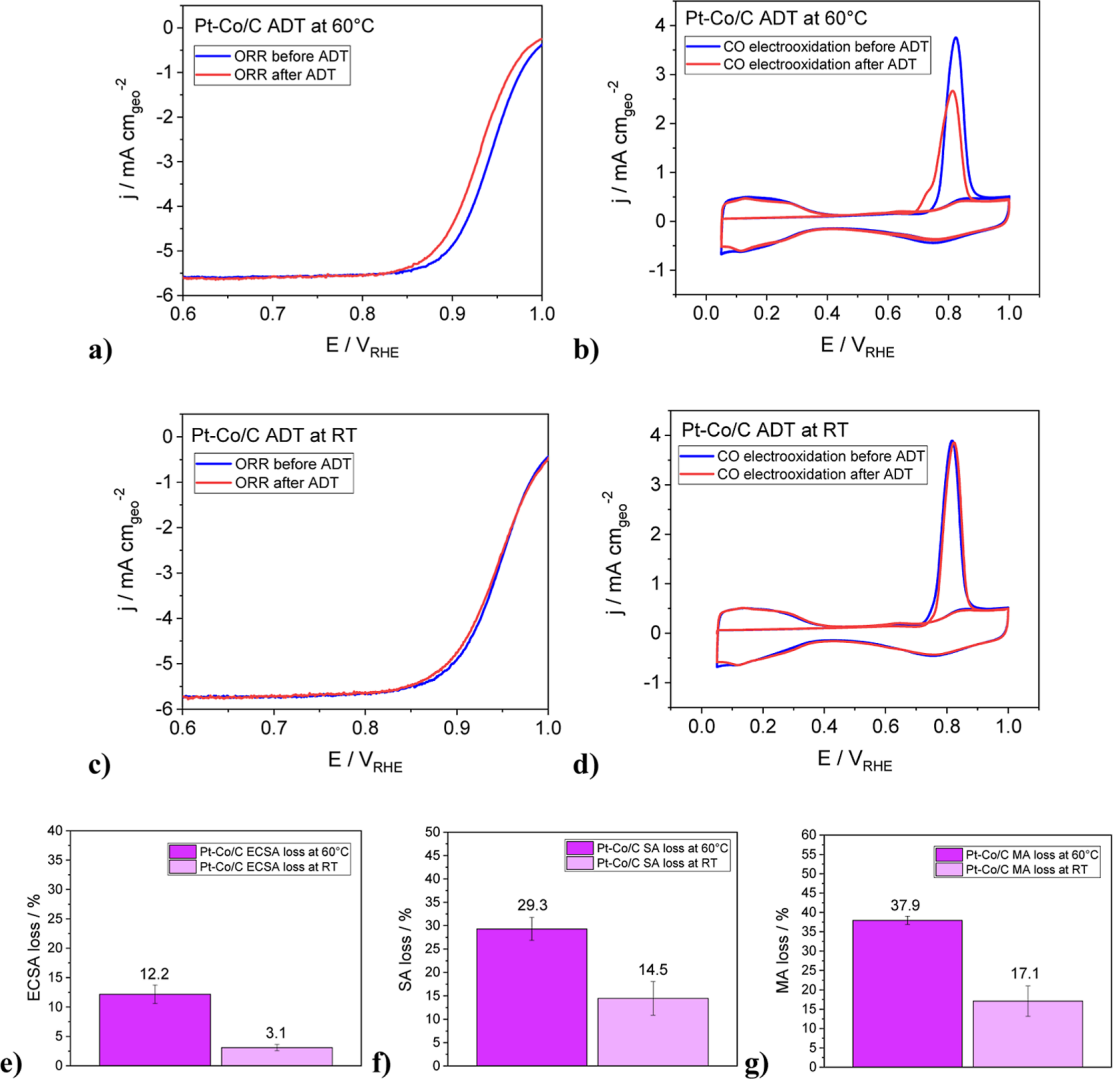
Comparison between Pt–Co/C (a,c) ORR anodic scans before and after the ADTs at 60 °C and RT with subtracted capacitive currents, performed from 0.05 to 1.00 V_RHE_, 20 mV s^–1^, at 1600 rpm, under O_2_ saturation; and (b,d) CO electrooxidation from 0.05 to 1 V_RHE_, 20 mV s^–1^, without rotation and under N_2_ saturation. The characterization protocols were carried out in a TF RDE setup (ex situ), at RT. Comparison between the losses in (e) ECSA, (f) SA and (g) MA after the ADTs performed at 60 °C and RT.

In CO electrooxidation measurements (Figure 7b), degradation is evident through the reduction in the area under the CO oxidation curve, signifying a decrease in the ECSA. The onset and the peak of the CO oxidation shifting toward more negative potentials, along with the formation of a small “shoulder” at approximately 0.75 V_RHE_, point to an increase in nanoparticle size and consequently a less uniform nanoparticle size dispersion,^74^ potentially resulting from agglomeration, coalescence and/or Ostwald ripening. This is supported by the previously discussed IL-STEM images (Figure 3a–d), which depict specifically the process of necking, coalescence and reshaping. From the similarities in the double layer capacitance region (0.4–0.6 V_RHE_) before and after the ADT, it could be concluded that relatively small degradation of the support took place, as the largest contribution to capacitive currents comes from the carbon support.^75^ This stability is to be expected since the upper potential limit of the degradation protocol (0.95 V_RHE_) does not allow for intense oxidation of carbon support,^76,77^ even at elevated temperatures. Negligible support degradation was also confirmed with the low-magnification IL-STEM imaging at both temperatures (Supporting Information Figures S1, S2, S8 and S9). Degradation at RT showcases similar trends during ORR measurements and CO electrooxidation, but to a much lesser extent (Figure 7c,d).

ECSA was determined by integrating the charge under the CO electrooxidation peak, while the SA and MA were calculated at 0.95 V_RHE_ using the Koutecký–Levich equation.^53^ The thin film electrochemical characterization results (Supporting Information Figure S14a–c) show beginning-of-test ECSA values of the commercial Pt–Co/C to be around 62.32 m^2^ g_Pt_^–1^, while SA equaled 0.1608 mA cm^–2^ and MA 0.1001 A mg^–1^. The intrinsic activity of Pt–Co/C is higher compared to pure Pt/C electrocatalysts due to the previously mentioned structural and/or electronic effects.^12–17^

The losses in electrochemical properties of the commercial Pt–Co/C sample after ADTs performed both at 60 °C and RT are depicted in Figure 7e–g. The losses in ECSA follow the morphological changes, including Ostwald ripening and/or coalescence of the nanoparticles shown. The sample retained approximately 88% of its ECSA after the ADT at 60 °C and 97% at RT, indicating a relatively low tendency for morphological restructuring via Pt oxidation and reduction under milder thermal conditions. This is consistent with the IL-STEM images (Figures 3a–h and 4a–d). Moreover, Jiang et al. showed in their 2020 study that Pt–Co/C, compared to pure Pt/C, demonstrates a smaller decrease in Pt–Pt and a smaller increase in Pt–O coordination number (CN) after an ADT, which points to a less intense restructuring and loss in active sites.^78^ This smaller loss in ECSA for the Pt–Co/C could be explained through the smaller tendency of Pt toward oxidation due to the decrease in the binding energy of oxygen to Pt, brought about by the strain, ligand and ensemble effects.^79,80^ Another parameter that affects the electrocatalyst’s tendency toward Pt oxidation is the Pt nanoparticle size. It has been shown in previous studies that smaller nanoparticles (1–4 nm diameter) are more prone to dissolution and then redeposition on bigger particles, a mechanism known as Ostwald ripening.^81–85^ Consequently, the relatively small loss in ECSA could also be due to the presence of larger particles, which could potentially reduce the likelihood of Ostwald ripening.

Following the ADTs, the sample exhibited a 15% SA loss at RT and 29% at 60 °C, thus the same rising trend regarding temperature. This larger loss of SA and its dependency on temperature could be attributed to the leaching of Co from the Pt–Co alloy nanoparticles, which is seemingly more pronounced at 60 °C.

This trend of increased Co dissolution with temperature was described by D̵ukić et al.^30^ in their 2022 paper, in which they analyzed the significance of both temperature and potential window on the stability of Pt-alloy electrocatalysts. By utilizing the EFC-ICP–MS setup, they showed that the Co dissolution follows Pt dissolution, and that the Co signal becomes higher with the increase in temperature, while the signal for Pt even seems to decrease.^30^ This lower Pt dissolution signal is explained through increased Pt redeposition at higher temperatures during the cathodic cycling and potential hold at the lower potential limit (LPL = 0.6 V_RHE_; Pt^2+^ + 2e^–^ → Pt_(s)_; *E*° = 1.2 V_RHE_), which is why the true Pt dissolution could have been masked.^86^ Moreover, due to its lower standard reduction potential (Co^2+^ + 2e^–^ → Co_(s)_; *E*° = −0.277 V_RHE_), Co does not redeposit during the cathodic scan and potential hold at the LPL. Accordingly, with the decrease in the amount of Co in the alloy nanoparticles, the synergistic interactions between Pt and Co are less pronounced, thus the portrayed loss.^87,88^

The effects of reduced ECSA (resulting from the increase in sphericity, increase in particle size, dissolution of smaller particles) and SA (decrease in Pt–Co interactions due to the dissolution of Co) are compounded in the case of MA, which is why we observe the highest percentage losses (Figure 7g). Based on previous comparisons, it can be concluded that the loss in MA for the Pt–Co/C largely resulted from the decrease in intrinsic activity due to Co dissolution and less so due to the reduction in active surface area, both of which are accelerated at higher temperatures.

It is important to note that degradation of Pt–Co/C has been shown to be different in aqueous half-cell setups compared to MEAs, especially at nanoparticle sizes relevant for practical applications.^89–91^ Consequently, additional optimization and testing should be done in order to more closely simulate degradation in PEMFCs, such as widening the degradation protocol’s potential window and increasing the concentration of Pt ions in the solution,^41^ along with the utilization of alternative cell designs, such as dry cell setups,^89,90,92^ or a modified floating electrode.^19,93^

## 4 Conclusions

In conclusion, by combining the HT-DE methodology with IL-STEM, nanoscale changes induced by the modified US DoE degradation protocol (fast potential cycling between 0.6 and 0.95 V_RHE_ for 10,000 cycles in 0.1 M HClO_4_) could be seen for the commercial Pt–Co/C electrocatalyst. The protocol was executed in a liquid half-cell at 60 °C and RT, which balances the throughput together with obtaining adequate information on the electrocatalyst durability and allows for additional observation on the influence of temperature on the nanoparticle structural changes.

From the low-magnification IL-STEM imaging after the ADT at 60 °C, it could be concluded that the sample was relatively stable. The outline of the carbon support was mostly unchanged, suggesting only minor carbon corrosion during the ADT. Under higher magnifications, substantial alterations in the structure of the Pt–Co nanoparticles were observed. Specifically, signs of intermediate nanoparticle coalescence mechanisms could be seen.

One of the mechanisms observed was the so-called “necking”, which represents the bridging of two nanoparticles that are sufficiently close and oriented toward each other with matching facets. The merging of the nanoparticles was accompanied by morphological changes brought about by the tendency for the minimization of the Pt–Co nanoparticle net surface energy. Additional intermediate mechanisms of coalescence observed included orientated attachment, along with total merging and final reshaping of nanoparticles.

Along with coalescence, dissolution and redeposition were also observed. We assume that both processes are likewise heavily influenced by the tendency for the minimization of nanoparticle surface energy. High-magnification IL-STEM images showed the transformation of Pt–Co nanoparticles to more thermodynamically stable, round-like shapes under oxide formation/reduction cycling. An increase in the circularity of the nanoparticle outline was confirmed through the size and circularity analysis of the Pt–Co nanoparticle IL-STEM images. Both dissolution and redeposition seemed to be contributing toward the increase in circularity. Comparatively, IL-STEM images taken after the ADT performed at RT show much smaller nanostructural changes, most notably a lack of necking and coalescence. Dissolution and redeposition are contributing to the trend of increased circularity of Pt–Co nanoparticles, but to a lesser extent than at 60 °C.

Electrochemical characterization of the commercial sample further elaborated the degradation behavior at both 60 °C and RT. Through CO electrooxidation measurements, degradation was evident through the reduction in the surface area under the oxidation curve, while the shifting of the onset and the peak toward more negative potentials, along with the formation of a small “shoulder”, pointed to an increase in nanoparticle size and consequently a less uniform nanoparticle size distribution. All these changes point to the loss in the ECSA, which can be explained through the particle coalescence (via either agglomeration and/or Ostwald ripening), increase in sphericity, particle size and the dissolution of smaller particles. On the other hand, the decrease in the SA could be attributed to the leaching of Co and the resulting decrease in Pt–Co interactions. All the mentioned mechanisms are compounded in the loss in MA, which is consequently the highest. Electrochemical measurements performed at RT revealed significantly smaller losses in the intrinsic properties of the electrocatalyst, further highlighting the role of temperature in driving degradation mechanisms such as Pt dissolution/redeposition and Co leaching. Since degradation of Pt–Co/C has been shown to be different in aqueous half-cell setups compared to MEAs, additional optimization and testing are necessary to more closely simulate degradation in PEMFCs, along with the utilization of alternative cell designs.

This work serves as an insight into the nanoscale degradation behavior of Pt–Co electrocatalyst in aqueous half-cells, and it accentuates the role of surface energy within this context. This is all done to improve the intrinsic properties of Pt-alloy-based electrocatalysts, especially the stability, through the fundamental understanding of the strengths and limitations of these materials at elevated temperatures.

## Supplementary Material



## Notes

Additional IL-STEM images at different magnifications for both temperatures; images with marked faceting, simulated XRD pattern; nanoparticle masks; Pt–Co/C cyclic voltammograms, ORR, Tafel and CO electrooxidation curves before and after ADTs performed at 60 °C and RT; Pt/C cyclic voltammograms, ORR, Tafel and CO electrooxidation curves before and after ADTs performed at 60 °C and RT; bar graphs comparing Pt–Co/C and Pt/C intrinsic properties and their losses after ADTs; comparison of XRD diffraction patterns, size and circularity analysis graph containing outlying particles (PDF)
