# Supplementary material for: Nanoscale Degradation Study of a Commercial Pt–Co/C Fuel-Cell Electrocatalyst at Elevated Temperature Utilizing Identical-Location Scanning Transmission Electron Microscopy
Source: J Phys Chem C Nanomater Interfaces. 2025 Aug 19;129(34):15419–32. doi: 10.1021/acs.jpcc.5c03548 (PMC12400420; doi:10.1021/acs.jpcc.5c03548)
Supplement: Supplementary file 1 [file jp5c03548_si_001.pdf]

Supporting information: Nanoscale Degradation Study of a Commercial Pt-Co/C Fuel-Cell Electrocatalyst at Elevated Temperature Utilising Identical-Location Scanning Transmission Electron Microscopy

Ante Matošin,[1,2] Lazar Bijelić,[1,3] Ana Rebeka Kamšek,[1,2] Goran Dražić,[1] Matija Gatalo,[1,4] Marjan Bele,[1] and Nejc Hodnik[1,3,5,*]

1 National Institute of Chemistry, Hajdrihova 19, 1001 Ljubljana, Slovenia

2 Faculty of Chemistry and Chemical Technology, Večna pot 113, 1000 Ljubljana, Slovenia

3 University of Nova Gorica, Vipavska 13, 5000 Nova Gorica, Slovenia

4 ReCatalyst, Hajdrihova 19, 1001 Ljubljana, Slovenia

5 Institute of Metals and Technology, Lepi pot 11, 1000 Ljubljana, Slovenia

** nejc.hodnik@ki.si (corresponding author)*

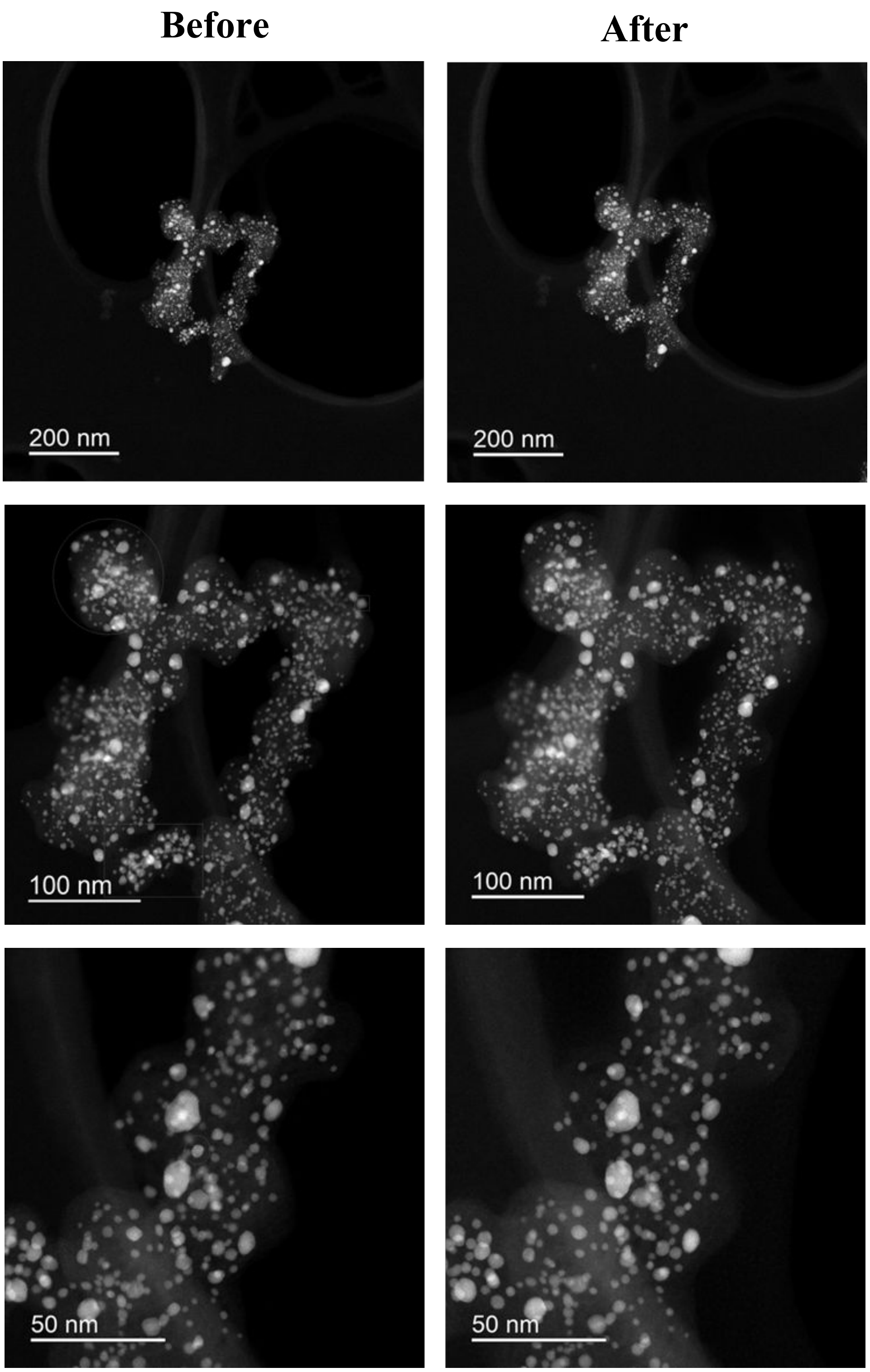

Figure S1. High-Angle Annular Dark Field (HAADF) IL-STEM images of the Umicore Pt-Co/C sample (Elyst Pt50 0690) at various magnifications. The images were taken before and after the ADT, which was performed in 0.1 M $HClO_4$ at 60 °C by potential cycling between 0.6 and 0.95 V_{RHE} for 10,000 cycles.

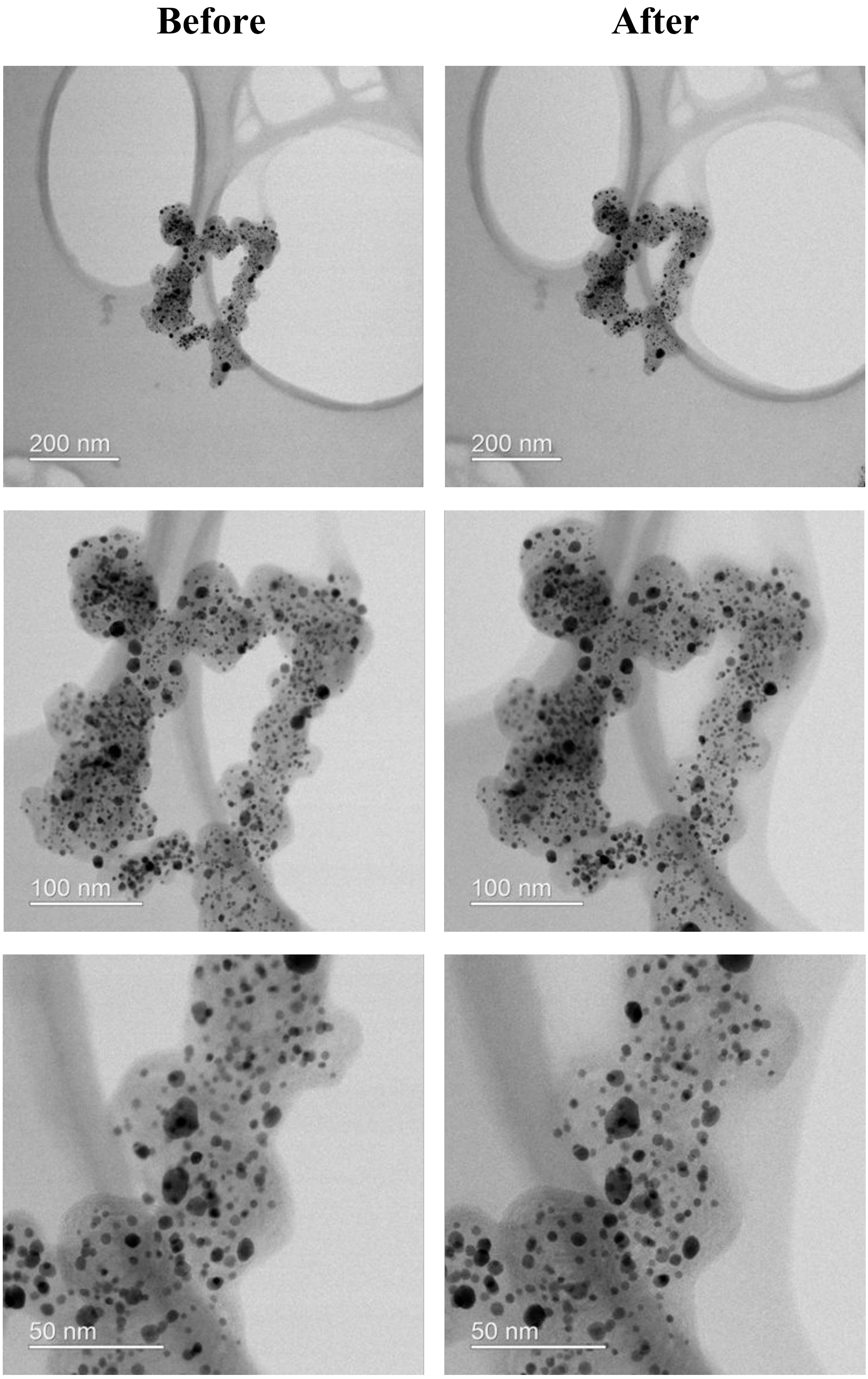

Figure S2. High-Angle Annular Bright Field (HAABF) IL-STEM images of the Umicore Pt-Co/C sample (Elyst Pt50 0690) at various magnifications after the ADT performed at 60°C.

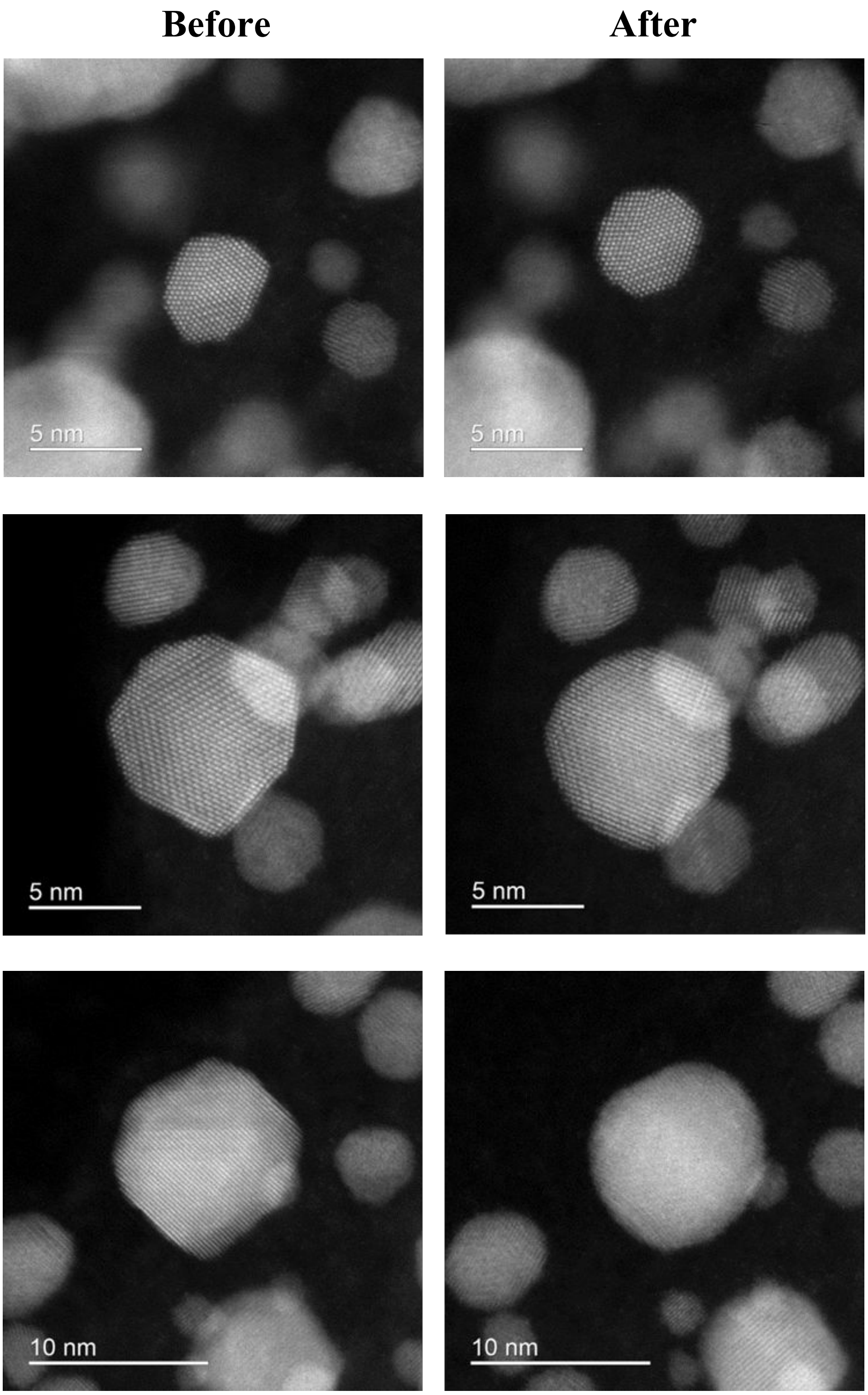

Figure S3. Additional High-Angle Annular Dark Field (HAADF) IL-STEM images of the Umicore Pt-Co/C (Elyst Pt50 0690) depicting dissolution and redeposition influenced by surface energy.

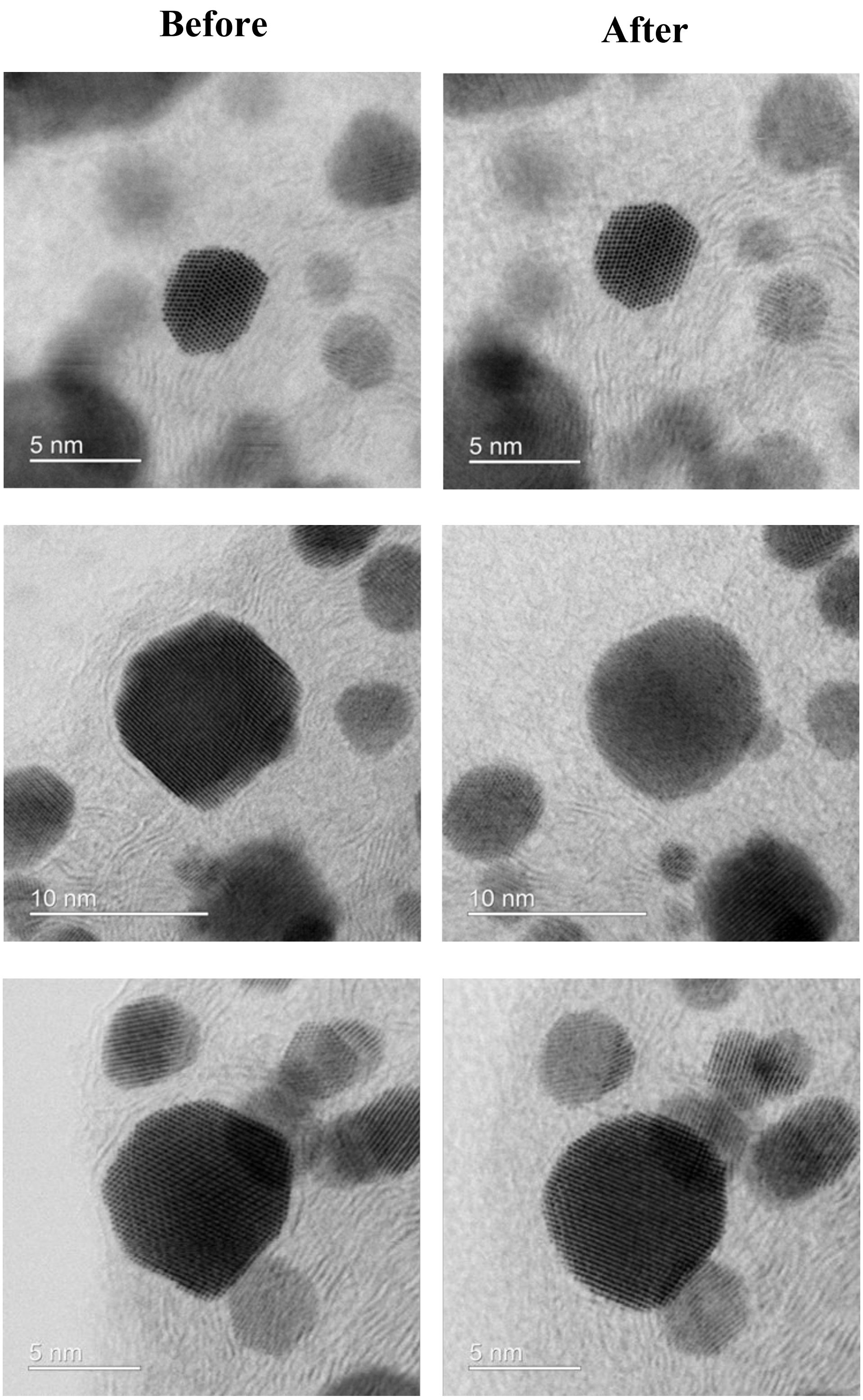

Figure S4. Additional High-Angle Annular Bright Field (HAABF) IL-STEM images of the Umicore Pt-Co/C sample (Elyst Pt50 0690) depicting dissolution and redeposition influenced by surface energy.

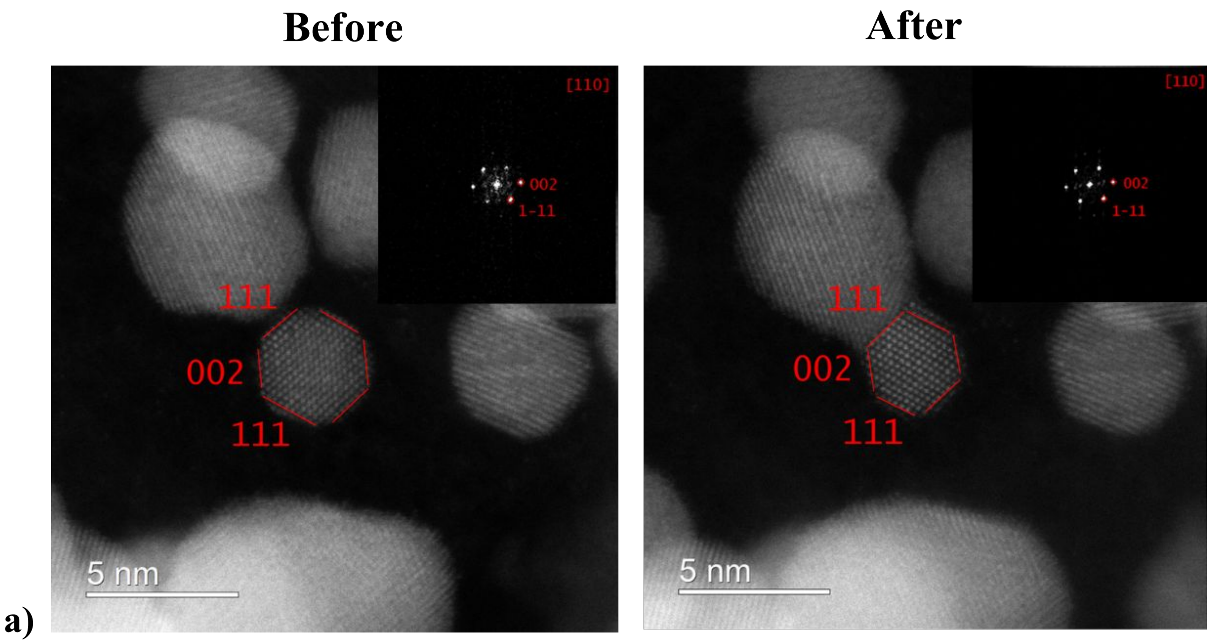

Figure S5. High-Angle Annular Dark Field (HAADF) IL-STEM images of the Umicore Pt-Co/C sample (Elyst Pt50 0690) with marked faceting on the nanoparticle undergoing necking.

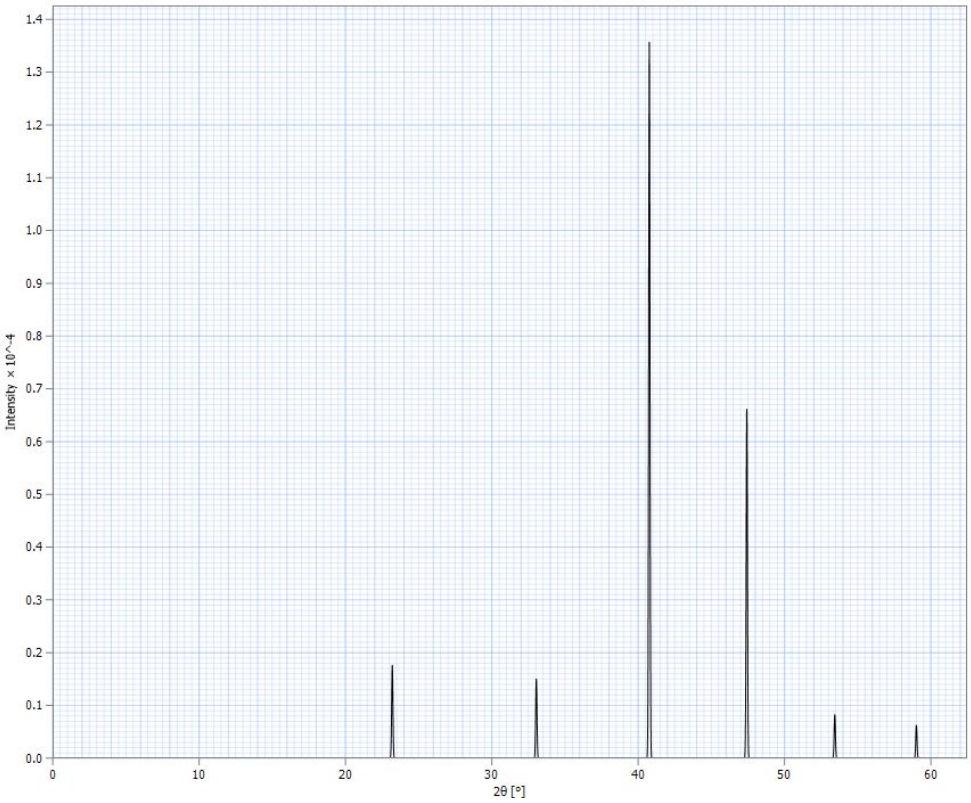

Figure S6. Simulated XRD pattern for Pt-Co P4/mmm using CrystalDiffract, crystal structure adapted from [https://doi.org/10.1051/jphys:01964002505060001].

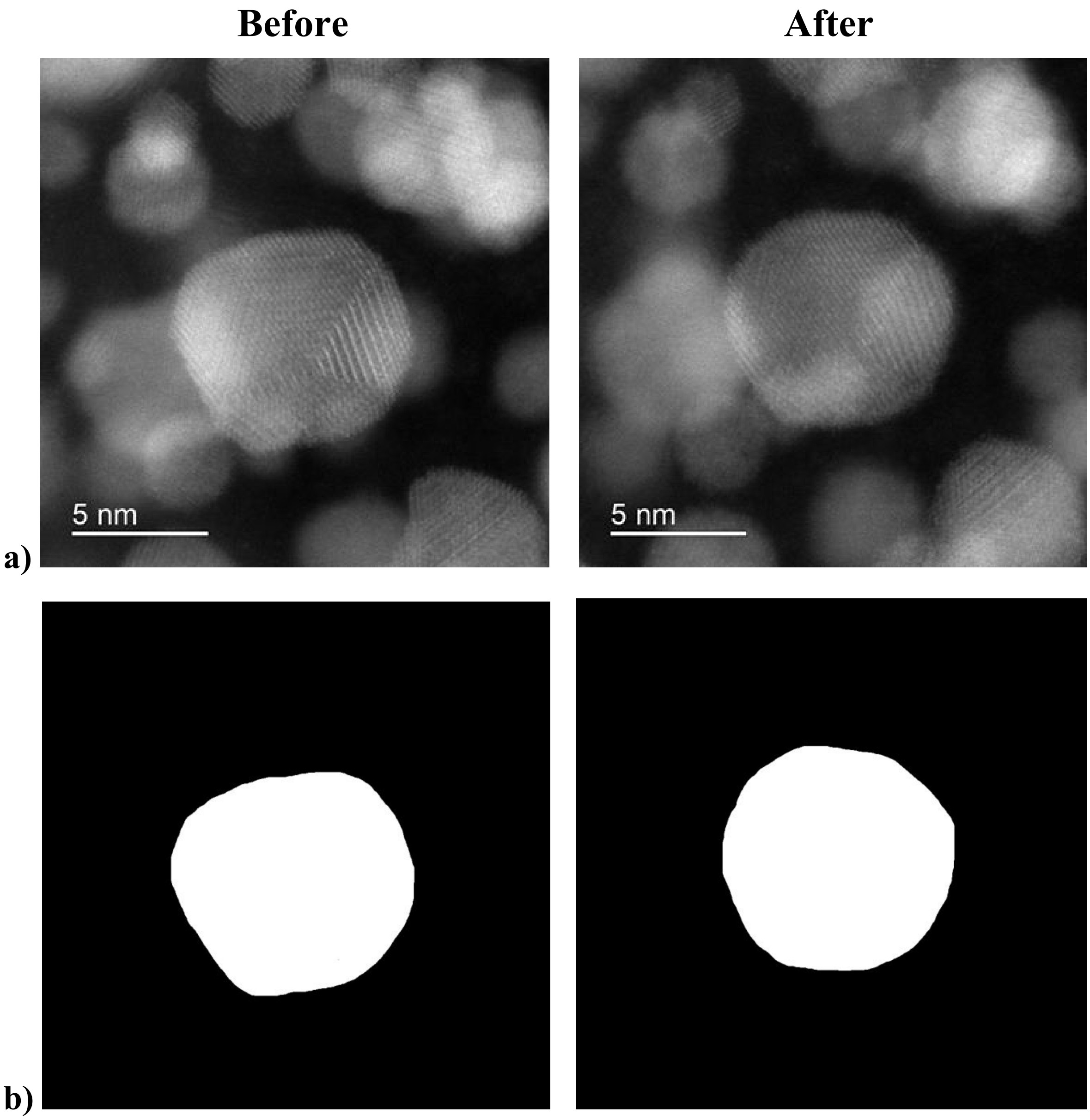

Figure S7. (a) High-Angle Annular Dark Field (HAADF) IL-STEM images of the Umicore Pt-Co/C sample (Elyst Pt50 0690) nanoparticle and (b) the corresponding nanoparticle mask. The masks were used to determine the change in circularity of nanoparticles after the ADT.

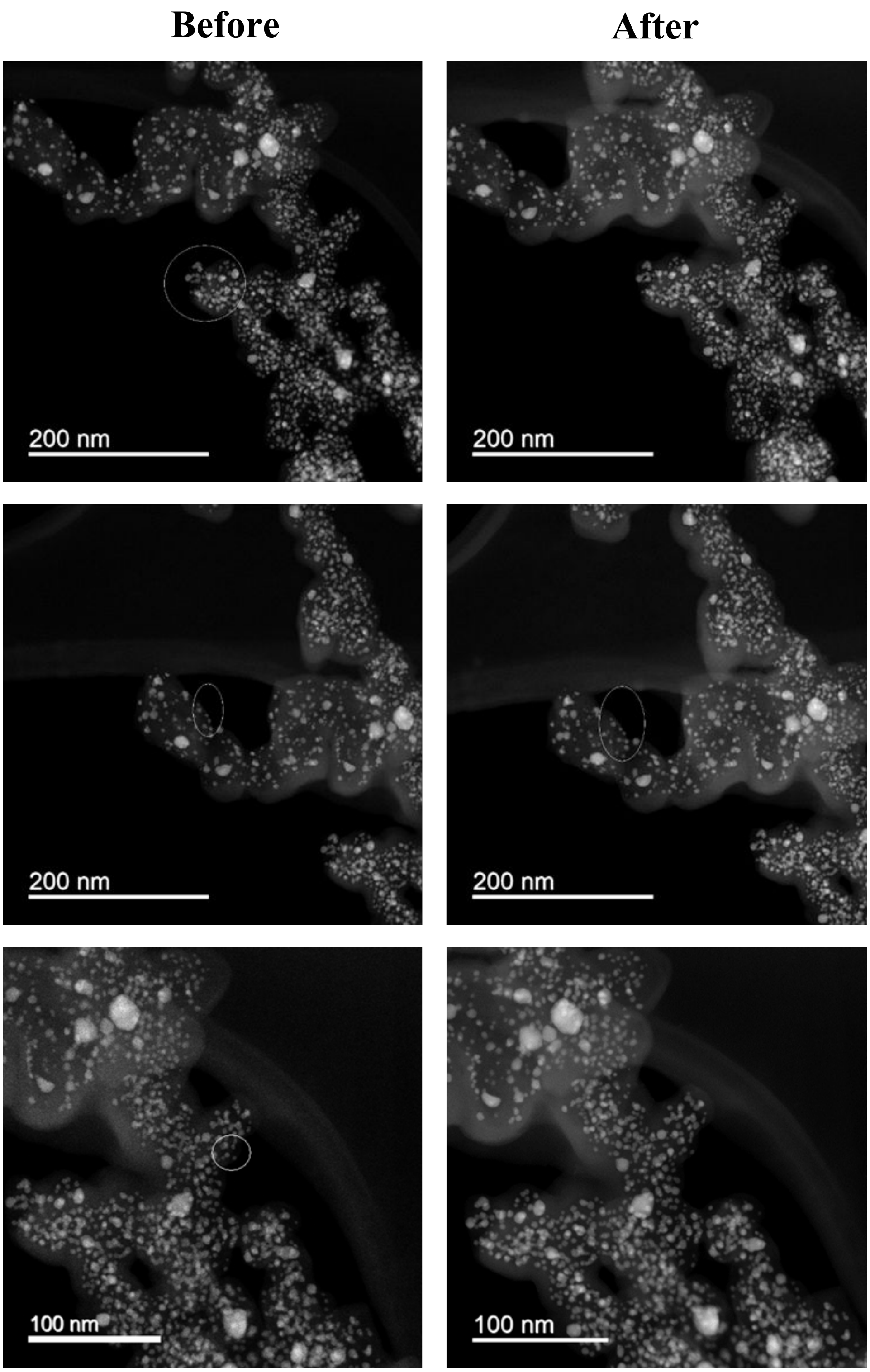

Figure S8. High-Angle Annular Dark Field (HAADF) IL-STEM images of the Umicore Pt-Co/C sample (Elyst Pt50 0690) at various magnifications. The images were taken before and after the ADT, which was performed in 0.1 M $HClO_4$ at RT by potential cycling between 0.6 and 0.95 V_{RHE} for 10,000 cycles.

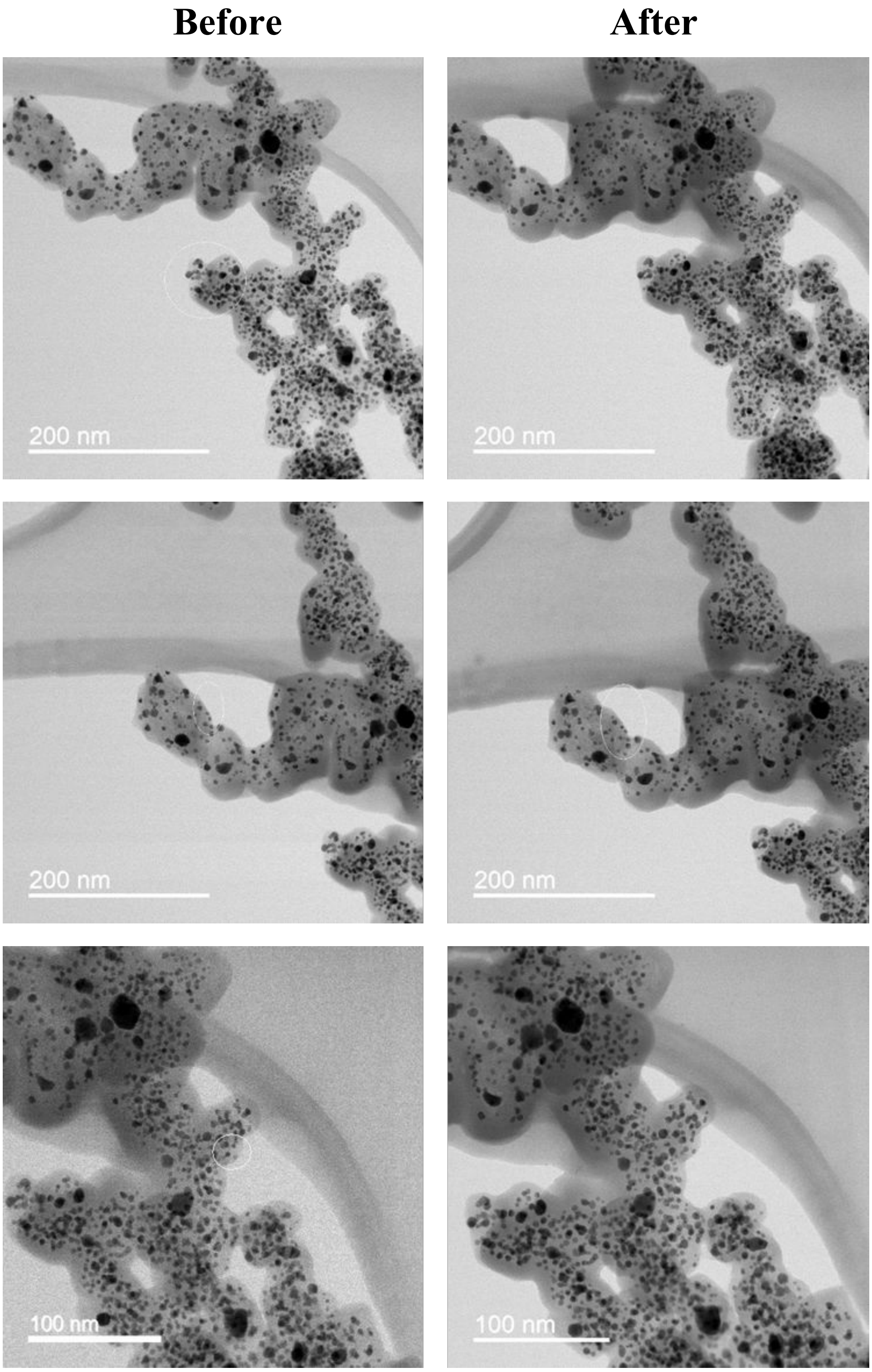

Figure S9. High-Angle Annular Bright Field (HAABF) IL-STEM images of the Umicore Pt-Co/C sample (Elyst Pt50 0690) at various magnifications after the ADT performed at RT.

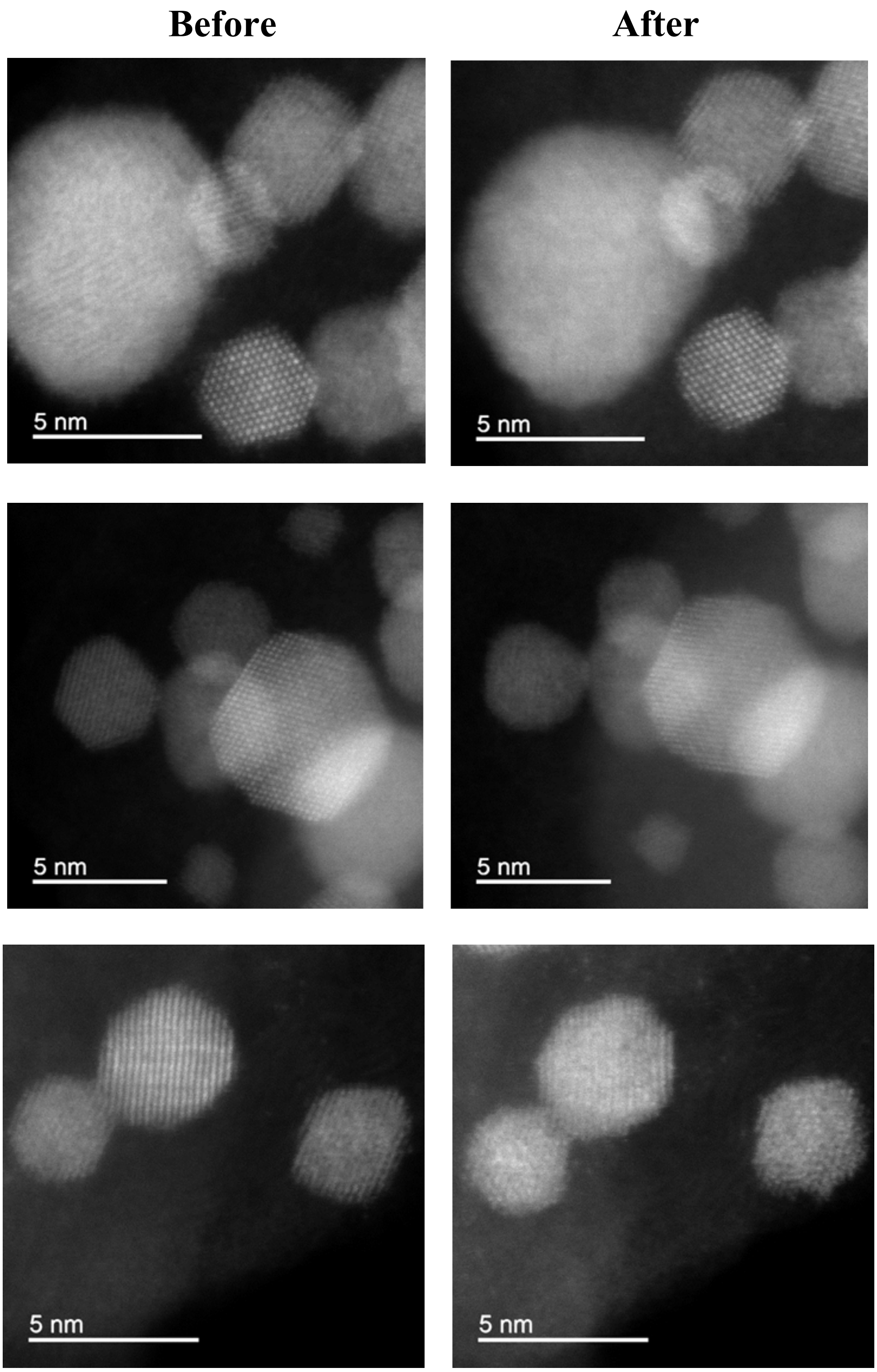

Figure S10. Additional High-Angle Annular Dark Field (HAADF) IL-STEM images of the Umicore Pt-Co/C (Elyst Pt50 0690) depicting dissolution and redeposition influenced by surface energy after the ADT performed at RT.

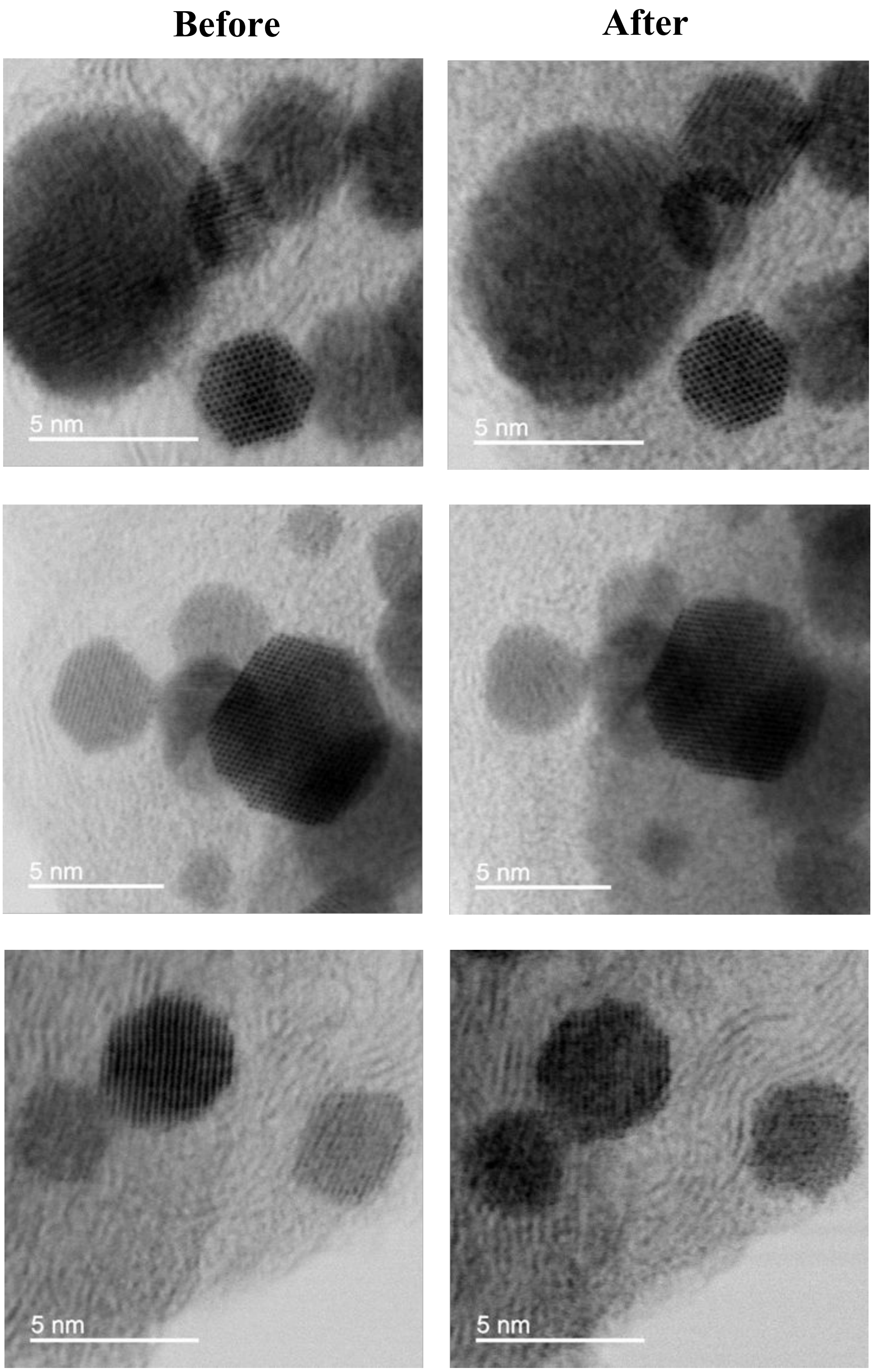

Figure S11. Additional High-Angle Annular Bright Field (HAABF) IL-STEM images of the Umicore Pt-Co/C sample (Elyst Pt50 0690) depicting dissolution and redeposition influenced by surface energy after the ADT performed at RT.

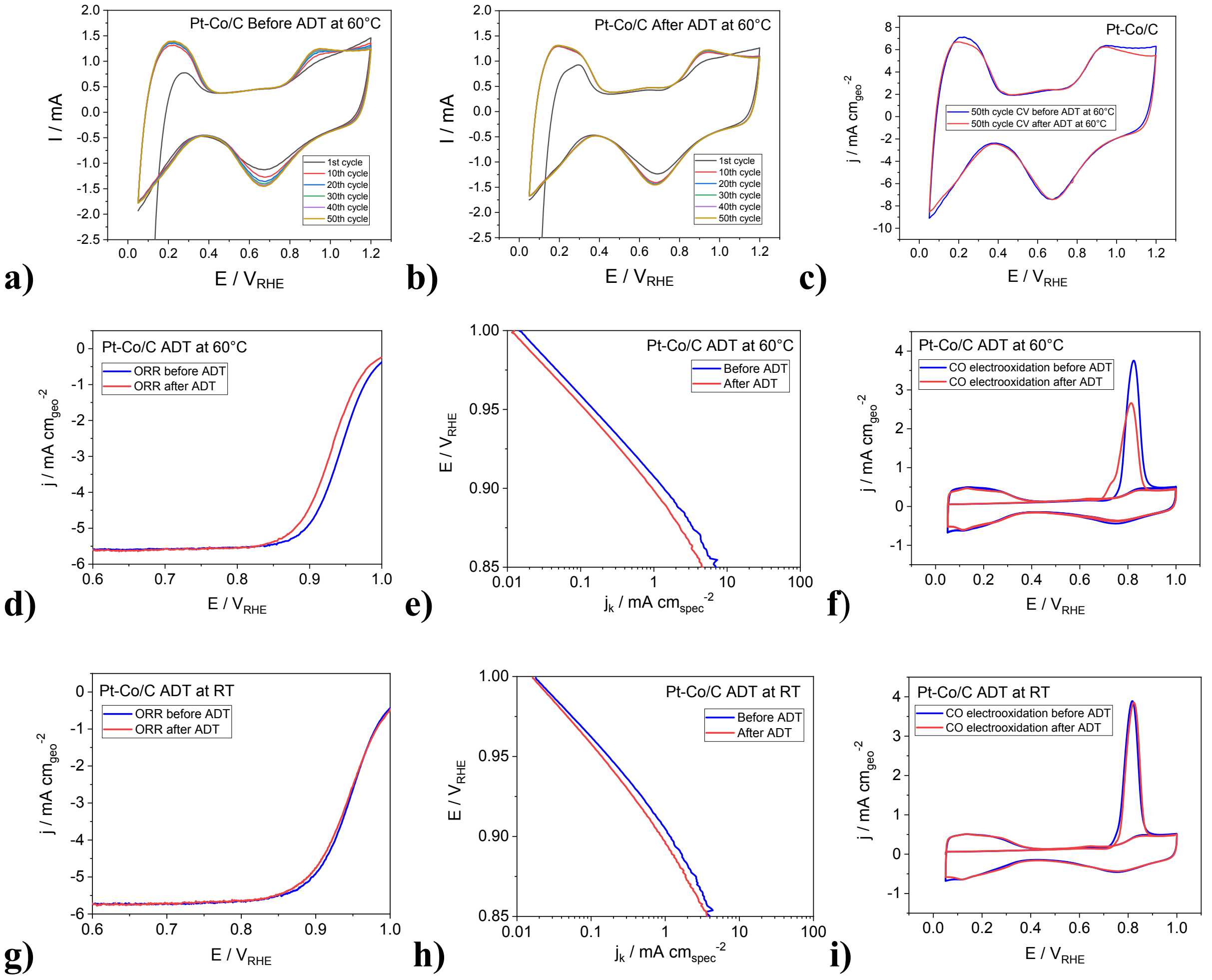

Figure S12. Cyclic voltammograms of the Pt-Co/C sample before (a), after (b) and comparison of the 50th CV before and after the ADT at 60°C (c). Cyclic voltammetry was done at room temperature, in 0.1M $HClO_4$ with argon purging, and it spanned 50 cycles from 0.05 to 1.2 V_{RHE}, with a scan rate of 300 mV s^{-1} and under a 600-rpm rotation rate. Comparison between Pt-Co/C (d) ORR anodic scans with subtracted capacitive currents, performed from 0.05 to 1.00 V_{RHE}, 20 mV s^{-1}, at 1600 rpm, under O_2 saturation; (e) the resulting Tafel plots; (f) CO electrooxidation from 0.05 to 1 V_{RHE}, 20 mV s^{-1}, without rotation and under N_2 saturation before and after the ADT performed at 60°C and room temperature (g-f).

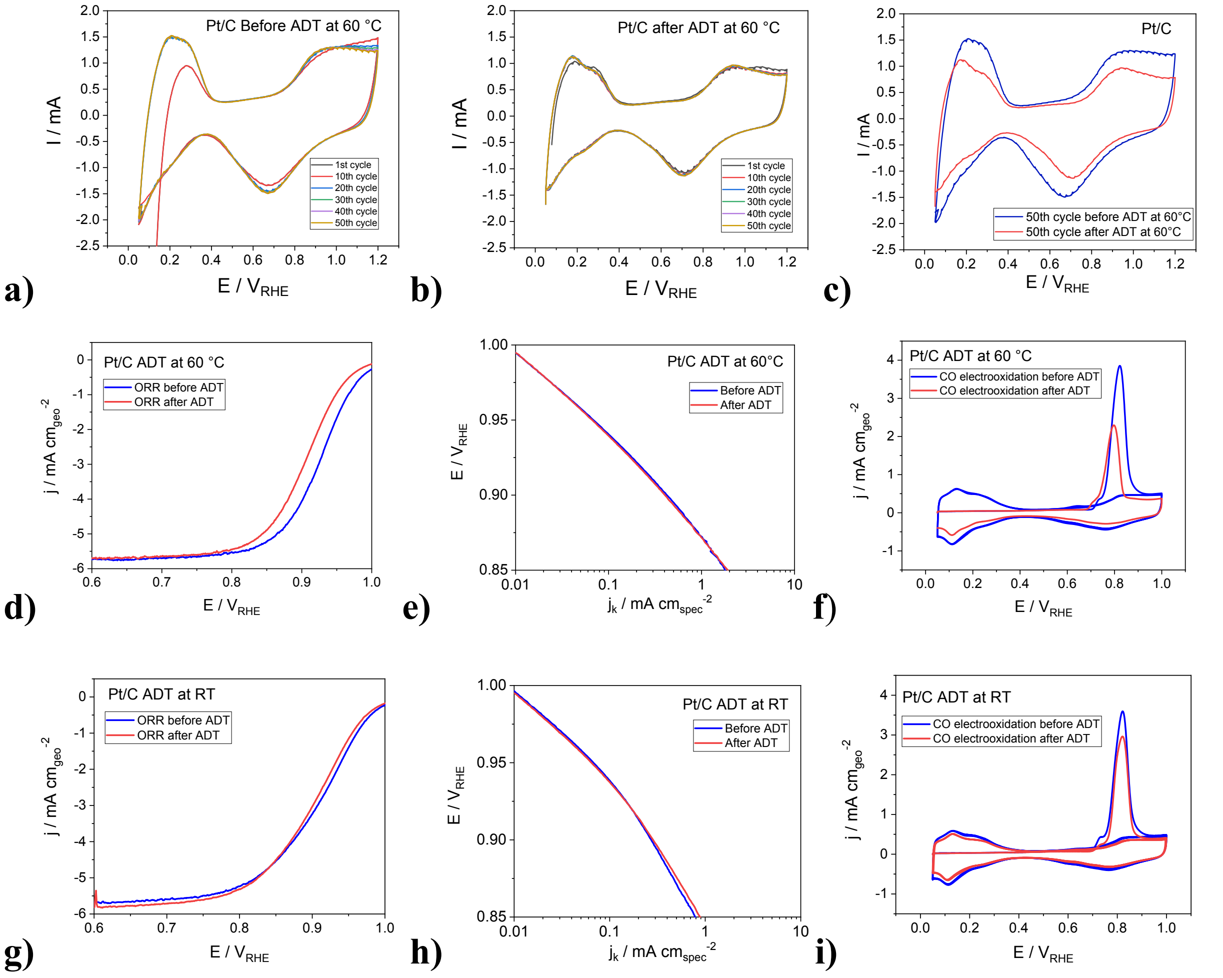

Figure S13. Cyclic voltammograms of the Fuel Cell Pt/C 40 wt. % Pt sample before (a), after (b) and comparison of the 50th CV before and after the ADT (c). Cyclic voltammetry was done at room temperature, in 0.1M $HClO_4$ with argon purging, and it spanned 50 cycles from 0.05 to 1.2 V_{RHE}, with a scan rate of 300 mV s^{-1} and under a 600-rpm rotation rate. Comparison between Pt-Co/C (d) ORR anodic scans with subtracted capacitive currents, performed from 0.05 to 1.00 V_{RHE}, 20 mV s^{-1}, at 1600 rpm, under O_2 saturation; (e) the resulting Tafel plots; (f) CO electrooxidation from 0.05 to 1 V_{RHE}, 20 mV s^{-1}, without rotation and under N_2 saturation before and after the ADT performed at 60°C and room temperature (g-f).

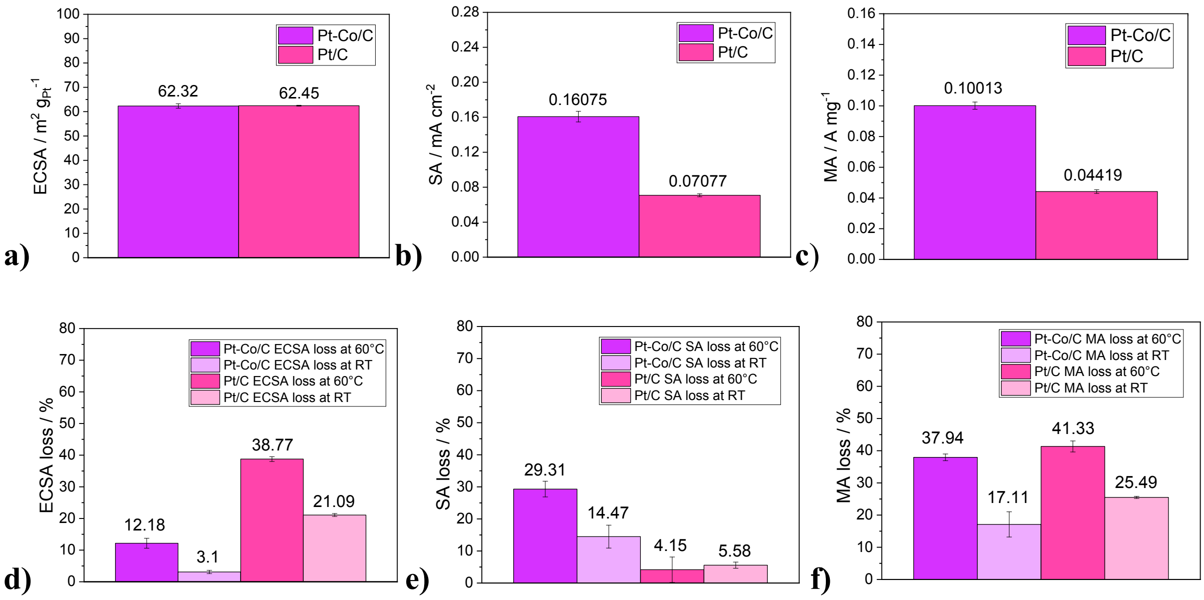

Figure S14. Comparison between the (a) ECSA, (b) SA and (c) MA of the Umicore (Elyst Pt50 0690) Pt-Co/C and Fuel Cell Pt/C 40 wt.% Pt sample, along with the losses (d-f) after the ADTs performed at 60°C and room temperature. The SA and MA values were determined at 0.95 V_{RHE}. The protocols were carried out before and after the ADT in a TF RDE setup (ex-situ), at room temperature. Platinum loading for the Pt-Co/C sample was 41.7 wt. % and 41.6 wt. % for Pt/C

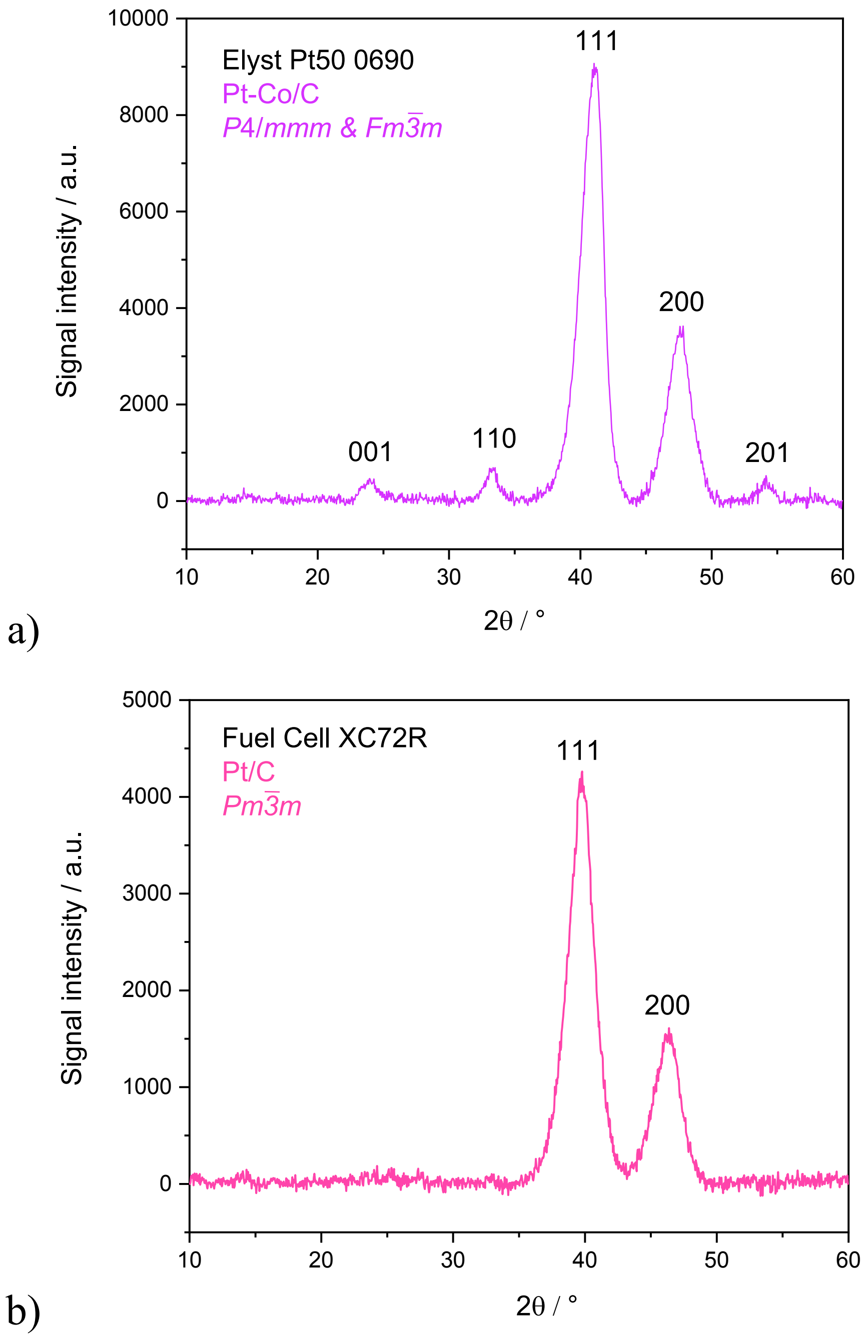

Figure S15. XRD diffraction patterns measured from the (a) Umicore Pt-Co/C sample (Elyst Pt50 0690) and (b) Fuel Cell Pt/C 40 wt.% Pt.

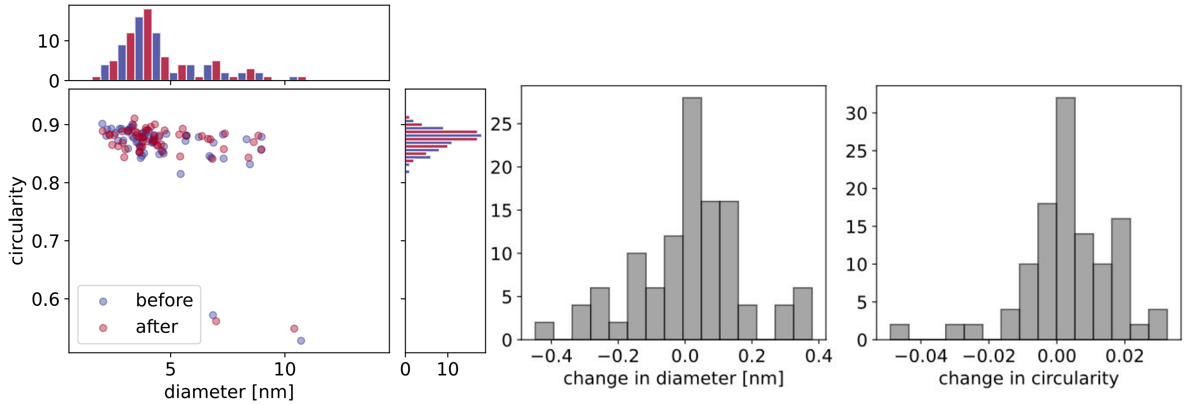

Figure S16. Size and circularity analysis before and after the ADT at RT, including two outlier particles with a circularity below 0.6. Changes in size and shape were determined and visualised through histograms to analyse the overall variations in size and circularity distributions.
